# Sheep (*Ovis aries*) T cell receptor alpha (TRA) and delta (TRD) genes and genomic organization of the TRA/TRD locus

**DOI:** 10.1186/s12864-015-1790-z

**Published:** 2015-09-18

**Authors:** Barbara Piccinni, Serafina Massari, Anna Caputi Jambrenghi, Francesco Giannico, Marie-Paule Lefranc, Salvatrice Ciccarese, Rachele Antonacci

**Affiliations:** Dipartimento di Scienze e Tecnologie Biologiche ed Ambientali, Universita’ del Salento, Lecce, Italy; Dipartimento di Scienze Agro-Ambientali e Territoriali, Universita’ degli Studi di Bari Aldo Moro, Bari, Italy; IMGT, Laboratoire d’ImmunoGénétique Moléculaire, Institut de Génétique Humaine, UPR CNRS 1142, Université Montpellier 2, 34396 Montpellier, Cedex 5 France; Dipartimento di Biologia, Universita’ degli Studi di Bari Aldo Moro, Bari, Italy

**Keywords:** T cell receptor, TRA/TRD locus, TRAV and TRDV genes, sheep genome, IMGT

## Abstract

**Background:**

In mammals, T cells develop along two discrete pathways characterized by expression of either the αβ or the γδ T cell receptors. Human and mouse display a low peripheral blood γδ T cell percentage ("γδ low species") while sheep, bovine and pig accounts for a high proportion of γδ T lymphocytes ("γδ high species"). While the T cell receptor alpha (TRA) and delta (TRD) genes and the genomic organization of the TRA/TRD locus has been determined in human and mouse, this information is still poorly known in artiodactyl species, such as sheep.

**Results:**

The analysis of the current *Ovis aries* whole genome assembly, Oar_v3.1, revealed that, as in the other mammalian species, the sheep TRD locus is nested within the TRA locus. In the most 5’ part the TRA/TRD locus contains TRAV genes which are intermingled with TRDV genes, then TRD genes which include seven TRDD, four TRDJ genes, one TRDC and a single TRDV gene with an inverted transcriptional orientation, and finally in the most 3’ part, the TRA locus is completed by 61 TRAJ genes and one TRAC gene.

Comparative sequence and analysis and annotation led to the identification of 66 TRAV genes assigned to 34 TRAV subgroups and 25 TRDV genes belonging to the TRDV1 subgroup, while one gene was found for each TRDV2, TRDV3 and TRDV4 subgroups. Multiple duplication events within several TRAV subgroups have generated the sheep TRAV germline repertoire, which is substantially larger than the human one. A significant proportion of these TRAV gene duplications seems to have occurred simultaneously with the amplification of the TRDV1 subgroup genes. This dynamic of expansion has also generated novel multigene subgroups, which are species-specific. *Ovis aries* TRA and TRD genes identified in this study were assigned IMGT definitive or temporary names and were approved by the IMGT/WHO-IUIS nomenclature committee.

The completeness of the genome assembly in the 3' part of the locus has allowed us to interpret rearranged CDR3 of cDNA from both TRA and TRD chain repertoires. The involvement of one up to four TRDD genes into a single transcript makes the potential sheep TRD chain much larger than any known TR chain repertoire.

**Conclusions:**

The sheep genome, as the bovine genome, contains a large and diverse repertoire of TRA and TRD genes when compared to the “γδ T cell low” species genomes. The composition and length of the rearranged CDR3 in TRD V-delta domains influence the three-dimensional configuration of the antigen-combining site thus suggesting that in ruminants, γδ T cells play a more important and specific role in immune recognition.

**Electronic supplementary material:**

The online version of this article (doi:10.1186/s12864-015-1790-z) contains supplementary material, which is available to authorized users.

## Background

T cell populations are characterised by two lymphocyte subsets that express distinct heterodimeric antigen-specific receptors (TRs) formed by alpha and beta chains (αβ T cells) or by gamma and delta chains (γδ T cells). For each chain, an antigen binding V domain is generated during lymphocyte development as a consequence of rearrangements of variable (V), diversity (D), and joining (J) genes. For the TR beta (TRB) and TR delta (TRD) chains, the V domain (V-beta and V-delta domain, respectively) results from a V-D-J rearrangement whereas for the TR alpha (TRA) and TR gamma (TRG) chains, the V domain (V-alpha and V-gamma, respectively) results from a V-J rearrangement. After transcription, the V**-**D-J or V-J rearranged sequence is spliced to the constant (C) gene. The TR genes are located in four discrete loci on chromosomes: TRA, TRB, TRG and TRD. The TRA and TRD genes are located at a single chromosomal location with the TRD locus nested within the TRA locus*.* As a consequence, the whole TRD locus is excised from the genomic sequence when a first TRA V-J rearrangement occurs in the TRA/TRD locus [1].

The genomic organization of the TRA/TRD locus is known in human and mouse. The human TRA/TRD locus spans 1.1 Mb and consists of an array of 54 TRAV genes belonging to 32–34 subgroups, with five V genes which have been found rearranged in both TRA and TRD chains (TRAV/DV genes) (IMGT®, http://www.imgt.org and IMGT/GENE-DB [1]). Two olfactory receptor genes are interspersed among the TRA genes: OR10G2 (located between TRAV1-1 and TRAV1-2) and OR4E2 (located between TRAV1-2 and TRAV2) [2, 3]. Four TRDV genes, with one of them located among the TRAV genes, three TRDD, four TRDJ and one TRDC genes, followed by a TRDV gene with an inverted transcriptional orientation, form the TRD locus nested within the TRA locus. At the 3’ end, 61 TRAJ genes and one TRAC gene complete the TRA locus [1].

The mouse TRA/TRD locus spans 1.7 Mb and is largely occupied by 132 TRAV genes including 9–10 TRAV/DV genes. Three olfactory receptor genes (Olfr1509, Olfr1508 and Olfr1507) are present between TRAV1 and TRAV2 genes. The TRD genes include, five TRDV, two TRDD, two TRDJ and one TRDC gene, followed by a TRDV gene with an inverted transcriptional orientation (IMGT®, http://www.imgt.org and IMGT/GENE-DB [4, 5]). At the 3' end of the locus there is a cluster of 60 TRAJ, followed by one TRAC gene.

In contrast to human and mouse, little is known about the genomic organization of the TRA and TRD loci in artiodactyls. The first analyses of artiodactyl TRA and TRD genes were predominantly based on chromosome mapping [6], cDNA and genomic clone analysis [7–10]. These analyses suggested that the general genomic organization of the TRA/TRD locus does not differ greatly from that of human and mouse, while a much larger repertoire of TRDV (particularly TRDV1) genes characterizes the TRD chains in sheep [10] as well as in other “γδ T cell high” species as cattle and pig [8, 11]. Due to the lack of full-scale genomic data, it was unclear whether the large number of TRDV1 sequences was the result of the existence of multiple genes or polymorphisms among animals. Different research groups have analysed the two cattle genomic assemblies [12, 13], released through public databases and continually being updated, in order to recognize the genomic organization of the TRA/TRD locus in cattle. Despite discrepancies in the total number of TRAV/TRDV genes, all data have confirmed that the cattle TRD chain repertoire is dominated by the expression of the multigene TRDV1 subgroup. As a matter of fact, Herzig et al. [14] by annotating the bovine Btau 3.0 assembly, have demonstrated the presence of 56 TRDV genes, 52 of which belong to the TRDV1 subgroup (with 46 and six of them functional and ORF, respectively). Two TRDV2 genes, and a single gene for both TRDV3 and TRDV4 subgroups were identified. Moreover, five TRDD, three TRDJ and one TRDC genes followed by a single TRDV5 gene in an inverted transcriptional orientation complete the bovine TRD locus. The same number of TRDV1 genes was predicted by Elsik et al. [13], based on an automated gene annotation of the Btau_4.0 genome assembly. In addition, they identified 71 functional TRAV, 38 TRAJ genes and a single TRAC gene within the TRA locus. At the same time, by manual annotation of the same Btau_4.0 assembly, Reinink and Van Rhijn [15] have detected more than one hundred TRDV genes and three hundred of TRAV or TRAV/DV genes. Most recently, the bovine assembly UMD3.1 was used to re-examine the genomic sequence of the entire TRA/TRD locus [16]. The analysis has identified 371 V genes in the TRA/TRD locus including 60 belonging to the TRDV1 subgroup, six TRDD, three TRDJ, one TRDC, 62 TRAJ and one TRAC, most of which located within a 3.5 Mb region of chromosome 10. The disparity of the data suggests that the definition of the total bovine TRA/TRD repertoire will be dependent on an improved genome assembly taking into account also the demonstrated high degree of heterozygosity in the bovine TRA/TRD locus [17], for which artifactual gene expansions cannot be excluded. However, although the exact number of TRA and TRD genes remains undetermined, all studies imply that a wide repertoire of TRAV and TRDV genes is present in the bovine genome.

In order to gain further insights into the genomic structure and the gene content of the TRA/TRD locus in “γδ high” species, we analysed the recent draft sheep genome assembly Oar_v3.1 [18]. Despite the fragmented and incomplete nature of the assembly, we have obtained important information on the sheep genomic TRA and TRD potential repertoire and its relationship with the expressed chain repertoires.

## Results

### Genomic organization of the sheep TRA/TRD locus

We employed the current Texel sheep (*Ovis aries*) whole genome assembly, Oar_v3.1, released by the International Sheep Genome Consortium (http://www.sheephapmap.org/) to NCBI (BioProject ID: 179263) to identify the TRA/TRD locus in this species. We retrieved a sequence of 1 Mb (gaps included) directly from the chromosome 7 genomic scaffold (NC_019464.1) that comprises the most 5’ variable gene of the TRA locus which is embedded among three olfactory receptor genes in conserved synteny with human and mouse, and the TRAC gene which, by analogy with all mammalian species studied so far, is located at the most 3’ end of the locus.

**Fig. 1:**
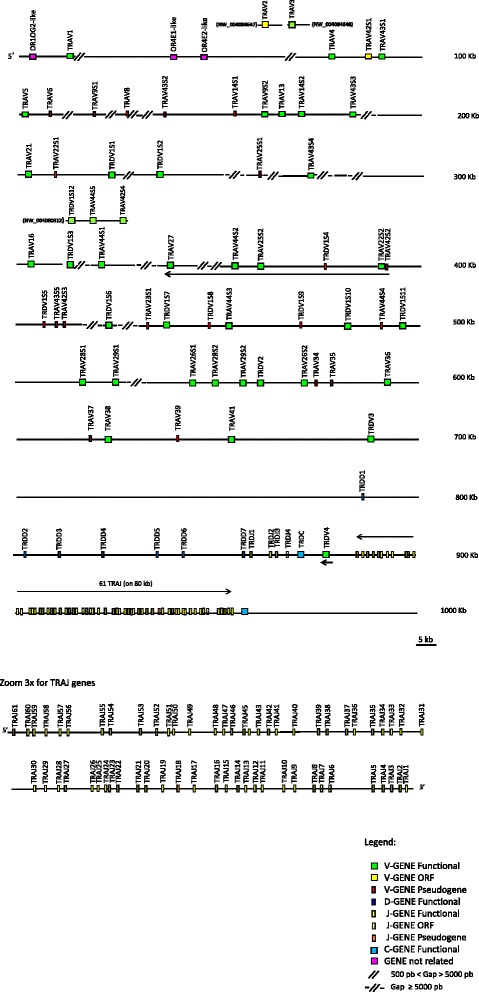
Sheep (*Ovis aries*) TRA/TRD locus. Schematic representation of the genomic organisation of the sheep TRA/TRD locus on chromosome 7 as deduced from the genome assembly Oar_v3.1. The unplaced genomic scaffolds NW_004080312, NW_004084646 and NW_004084647 are inserted in the map in a potential localization (see text). The diagram shows the position of all related and no-related TRA/TRD genes according to nomenclature. Boxes representing genes are not to scale. Exons are not shown. Arrows indicate transcriptional orientation of the V genes. The arrow above the line of the TRAJ genes indicates the 80 kb region that has been magnified in the lower part of the figure

All TRA/TRD genes within the assembly were identified and annotated using both the human sequence as a reference and the sheep cDNA and genomic clones collections [9, 10]. We also utilized the homology-based method, aligning the sheep retrieved sequence against itself with the PipMaker program [19] (Additional file 1). The presence in the pip of superimposed lines clearly indicates the occurrence of redundant matches between sheep genes (especially TRAV and TRDV genes), due to the homology among the different groups of genes. All sheep genes were classified as TRA or TRD on the basis of the percentage of nucleotide identity with the corresponding mammalian genes available in the GEDI (for GenBank/ENA/DDBJ/IMGT/LIGM-DB) database. The beginning and end of each coding exon was then identified with accuracy by the presence of splice sites or flanking recombination signal (RS) sequences of the V, D, and J genes. The analysis of the genome sequence revealed that, as in the other species of mammals, the sheep TRA/TRD locus has a similar organization with the TRD locus nested within the TRA locus and includes, from 5’ to 3’, the TRAV genes (43 genes), intermingled with TRDV genes (13 genes), seven TRDD, four TRDJ genes and one TRDC gene, followed by a single TRDV gene with an inverted transcriptional orientation (Fig. 1). At the 3’ end, the locus is completed with 61 TRAJ genes and one TRAC gene. Moreover, additional 23 TRAV and 14 TRDV genes were identified on a total of 24 unplaced genomic scaffolds*,* which are assumed to be located within the locus on chromosome 7 (Table 1). Hence, the sheep TRA/TRD locus extends an additional 280 kb.

**Table 1:** Sheep TRA/TRD genes on unplaced genomic scaffolds. The GEDI ID and the size of each scaffold is reported

NW_number	length (bp)	TRV gene name
A (scaffolds inserted in the map)		
004080312	20236	TRDV1S12, TRAV44S5, TRAV3S4
004084646	5428	TRAV3
004084647	5868	TRAV2
B (scaffolds not inserted in the map)		
004080344	6398	TRAV29S1
004080351	2921	TRAV22S3
004080352	6415	TRDV1S13
004080507	8138	TRAV17
004080664	6480	TRAV9S3
004080708	3005	TRAV22S4
004080772	15309	TRAV45S1, TRAV45S2
004080962	5683	TRAV12
004081110	23618	TRAV22S5, TRDV1S14, TRAV22S6
004081259	10995	TRDV1S15
004081534	24786	TRAV18S2, TRAV45S3, TRAV45S4
004081794	11900	TRAV10
004081954	3251	TRDV1S16
004082010	15522	TRDV1S17
004082156	2582	TRDV1S18
004082203	5855	TRDV1S19
004082507	54299	TRAV23S2, TRDV1S20, TRDV1S21, TRAV44S6, TRDV1S22, TRAV44S7, TRDV1S23
004082570	2995	TRAV9S4
004082847	15903	TRAV45S5
004083226	13533	TRDV1S24
004083566	7770	TRDV1S25

### Classification of the TRA and TRD genes

The TRAV and TRDV genes located on the Oar_v3.1 Chr7 genomic assembly were analysed together with the genes identified on the unplaced genomic scaffolds since it is expected that, like in the other mammalian species, these genes are all located within the TRA/TRD locus which in sheep is on chromosome 7. Consequently, a provisional nomenclature was assigned according to IMGT®, the international ImMunoGeneTics information system® (IMGT®, http://www.imgt.org, [20]), and IMGT® nomenclature [21]. Particularly, within a TR V subgroup, each gene was numbered first in the locus on chromosome 7 and then, in the provisional ordinated unplaced scaffolds. Additional file 2 summarizes the identified TRA/TRD genes. The TRAV and TRDV subgroups were established adopting the criterion that sequences with nucleotide identity of more than 75 % in the V-region belong to the same subgroup.

We have annotated 66 TRAV germline genes, which could be assigned to 34 distinct subgroups. Their functionality was defined, based on the IMGT rules as described at http://www.imgt.org/IMGTScientificChart/SequenceDescription/IMGTfunctionality.html [21]. Forty-three genes were predicted to be functional (about 65 %), five genes ‘ORF’ and 18 pseudogenes (Additional file 3). In order to classify the sheep TRAV gene subgroups, we performed a phylogenetic analysis combining, in the same alignment, the V-REGION nucleotide sequences of the sheep TRAV genes with those of the human TRAV genes. All functional, ORF and pseudogenes (except ten sheep TRAV pseudogenes with frameshifts and the human TRAV8-5) were included in the analysis. An unrooted phylogenetic tree was made using NJ method [22] (Additional file 4). The tree shows that 29 sheep subgroups form a monophyletic group with a corresponding human gene subgroup, consistent with the occurrence of distinct subgroups prior to the divergence of the two mammalian species. Therefore, in accordance with the phylogenetic clustering, we classified each of these sheep TRAV subgroups as orthologues to their corresponding human subgroups.

**Fig. 2:**
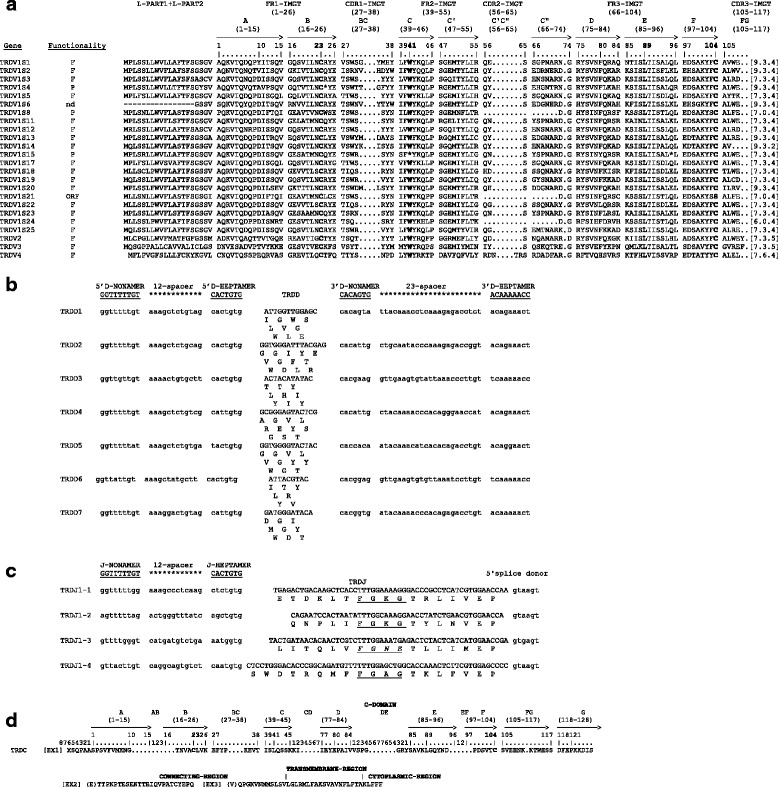
TRD genes. In a, the IMGT Protein display of the sheep TRDV genes. Only functional genes, ORF and in-frame pseudogenes are shown. The description of the strands and loops and of the FR-IMGT and CDR-IMGT is according to the IMGT unique numbering for V-REGION [27]. The CDR-IMGT AA lengths are indicated in square brackets. nd: not defined (indicates that the AA sequence of the TRDV1S6 gene is incomplete and its functionality cannot be defined). In b and c, nucleotide and deduced AA sequences of the sheep TRDD and TRDJ genes. The consensus sequences of the heptamer and nonamer [41] are provided at the top of the figure and underlined. The numbering adopted for the gene classification is reported on the left of each gene. In **b**, the AA sequences of the TRDD genes in the three coding frames are reported. In **c**, the donor splice site for each TRDJ is shown. The canonical FGXG motifs are underlined. The unusual TRDJ1-4 gene motif is in italics. In d, IMGT Protein display of the TRDC gene. Description of the strands and loops is according to the IMGT unique numbering for C-DOMAIN [42]

Interestingly, it is of note the existence of four sheep-specific subgroups, mostly with several members, probably as a result of recent duplications in the ovine TRA locus. These additional subgroups have been numbered with TRAV42 and above (subgroups boxed in Additional file 4). In contrast, 12 human TRAV subgroups were not found in sheep (genes boxed in Additional file 4). Finally, in order to classify the sheep TRAV pseudogenes with frameshifts (which were not included in the tree), we analysed their V region sequences with the IMGT/V-QUEST tool [23, 24] using the option ‘Search for insertions and deletions in V-REGION’ and aligned with the germline human TRAV sequence set from the IMGT reference directory. In this way, ten sheep pseudogenes with frameshifts could be classified as TRAV22S1, TRAV22S4, TRAV23S2, TRAV34, TRAV42S2, TRAV42S3, TRAV43S2, TRAV43S5, TRAV44S4 and TRAV44S7 (Additional file 2 and Additional file 3). Altogether this TRAV gene analysis demonstrates that 30 out of 34 sheep subgroups are shared with humans. In Table 2, the total sheep TRAV germline repertoire is reported in comparison with the human one. It is interesting how the orthology between human and sheep genes may guide the positioning of some sheep unplaced scaffolds within the Oar_v3.1 Chr7 genome sequence, in a syntenic region of the TRA/TRD locus. As an example, the sheep TRAV3 and TRAV2 genes recognised respectively in the NW_004084646 and NW_004084647 unplaced scaffolds (Table 1), could be tentatively positioned at one of the two gaps present at the 5’ part of the TRA/TRD locus (Fig. 1).

**Table 2:** The sheep and human germline TRAV repertoires

Subgroups	Sheep genes			Human genes		
	F	ORF	P	F	ORF	P
TRAV1	1			2		
TRAV2		1		1		
TRAV3	1			(1*)		(1*)
TRAV4	1			1		
TRAV5	1			1		
TRAV6			1	6		
TRAV7				1		
TRAV8			1	5	1	1
TRAV9	3		1	2		
TRAV10	1			1		
TRAV11				1		
TRAV12	1			3		
TRAV13	1			2		
TRAV14/DV4	1	1		1		
TRAV15						1
TRAV16	1			1		
TRAV17	1			1		
TRAV18	2			1		
TRAV19				1		
TRAV20				1		
TRAV21	1			1		
TRAV22	4	1	1	1		
TRAV23/DV6			2	1		
TRAV24				1		
TRAV25	1		1	1		
TRAV26	2			2		
TRAV27	1			1		
TRAV28	2			1		
TRAV29/DV5	2			(1*)		(1*)
TRAV30				1		
TRAV31						1
TRAV32						1
TRAV33						1
TRAV34			1	1		
TRAV35			1	1		
TRAV36/DV7	1			1		
TRAV37			1			1
TRAV38/DV8	1			2		
TRAV39		1		1		
TRAV40				1		
TRAV41	1			1		
TRAV42	1	1	2			
TRAV43	3		2			
TRAV44	4	2	1			
TRAV45	4		1			
TOTAL	43	7	16	43(+2*) 1	8(+2*)	

*Functional or Pseudogene

The definition of the four TRDV subgroups and the adopted nomenclature are consistent with previous reports [10, 25]. Eight out of 28 TRDV genes (about 28 %) have been predicted to be ORF or pseudogenes (Additional file 3), even if it is possible that the total number of V pseudogenes is slightly underestimated since the full-length coding sequence of some genes is not available (Additional file 2). However, a phylogenetic analysis was performed to confirm the membership of the TRDV1S6, TRDV1S8 and TRDV1S16 genes to the TRDV1 multimember gene subgroup since the level of nucleotide identity among them and between some but not all the other members is less than 75 % (data not shown). Thus, the nucleotide coding sequences (L-PART1 + V-EXON) of all sheep TRDV genes were combined in the same alignment and an unrooted tree was made using NJ method [22] (Additional file 5). In the tree, the membership of the TRDV1 genes is clearly supported by the monophyletic grouping. Besides, it is worth noting the high level of nucleotide identity (>97 %) between the TRDV1S3 and TRDV1S12 as well as the TRDVS10 and TRDVS18 coding regions as these sequences may represent allelic variants of the same gene [10]. The high percentage of nucleotide identity observed also within the flanking and intronic sequences confirmed that the TRDV1S3 and TRDV1S12 genes could represent the same gene indicating the occurrence of a redundant sequence in the genome assembly obtained from two individuals (a male and a female Texel sheep). More precisely, it is possible that the entire unplaced scaffold NW_004080312 containing the TRDV1S12 gene could be inserted within the Oar_v3.1 Chr7 genome sequence which would result in a merge of the TRDV1S12 and TRDV1S3 gene models (Fig. 1, Table 1). On the contrary, TRDV1S10 and TRDV1S18 have to be considered distinct genes due to the low homology especially within the intronic sequence (data not shown).

Genomic locations and classification of the sheep TRDD, TRDJ and TRAJ genes were also determined (Additional file 2). They were annotated and classified, according to the international nomenclature (IMGT®, http://www.imgt.org, [4, 20]) with a number corresponding to their position from 5’ to 3’ within the locus. Seven TRDD genes were localised in a region spanning about 153 kb between TRDV3 and TRDJ1 (Fig. 1). To date, five and at least six TRDD genes have been demonstrated in bovine and pig TRD locus, respectively [14, 26]. In contrast to a previous report [9] but consistent with human and pig (IMGT®, http://www.imgt.org; [26]), four TRDJ genes are located upstream of the TRDC gene. Similarly to human and pig, at least 61 TRAJ genes lie in the genomic region between TRDC and TRAC (Fig. 1 and Additional file 2).

### Structure analysis of the TRA and TRD genes

The deduced amino acid (AA) sequences of the sheep TRAV genes were manually aligned according to IMGT unique numbering for the V-REGION [27] to maximize percentage of identity (Additional file 6). Only potential functional genes and in-frame pseudogenes are shown. All sequences exhibit the typical framework regions (FR) and complementarity determining regions (CDR) and the four amino acids: cysteine 23 (1st-CYS) in FR1-IMGT, tryptophan 41 (CONSERVED-TRP) in FR2-IMGT, hydrophobic AA 89, and cysteine 104 (2nd-CYS) in FR3-IMGT [27]*.* The TRAV gene subgroups show a high structural diversity both in AA compositions and in length. In particular, the CDR1-IMGT is five, six or seven AA long. The CDR2-IMGT ranges in length from four to eight AA and the germline CDR3-IMGT is two to four AA long. Also the FR1-IMGT is 25 or 26 AA long.

**Fig. 3:**
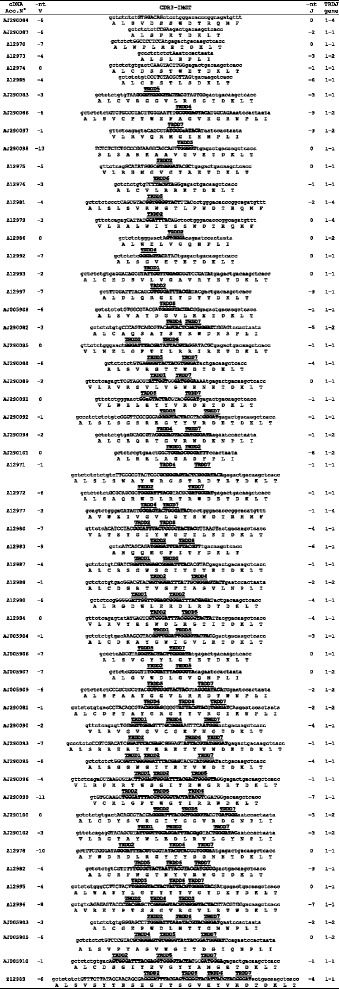
CDR3 nucleotide and predicted AA sequences retrieved from the TRD cDNA clones. The first column reports the accession numbers of all cDNA sequences. CDR3-IMGT sequences are shown from codon 105 (codon after the 2nd-Cys 104 of the V-REGION) to codon 117 (codon before J-PHE 118 of the J-REGION) according to the IMGT unique numbering [27]. They are grouped on the basis of the absence or the presence of one, two, three and four TRDD genes. Nucleotides of the 3’V-REGION and of the 5’J-REGION are indicated in lower cases. The sequences considered to present recognisable TRDD genes are indicated in bold upper cases with the indication of the TRDD gene name. Nucleotide substitutions with respect to the germline TRDD sequence are indicated in bold lower cases. Nucleotides that cannot be attributed to any V, D or J regions (N-nucleotides) are indicated in capital letters on the left and on the right side, and in between of the TRDD regions. Numbers in the left and right columns indicate the number of nt that are trimmed from the 3’V-REGION and 5’J-REGION, respectively. The last column on the right indicates the TRDJ genes

The nucleotide and deduced AA sequences of the 61 TRAJ genes identified in the genome assembly are reported in Additional file 6. 56 TRAJ genes were assessed as functional genes and they conserve the canonical F/W–G–X–G amino acid motif (where F is phenylalanine, W tryptophan, G glycine, and X any AA) (IMGT Repertoire, http://www.imgt.org, [27, 28]). Each TRAJ gene is flanked by the J-RS at the 5' end, and a donor splice at the 3' end. Two TRAJ genes were defined as ORF and three as pseudogenes.

The sheep TRAC exon-intron organization was determined (Additional file 6). The TRAC gene consists of three translated exons and a fourth untranslated exon. The first, second and third exons are 273, 45 and 108 bp in length, respectively. The fourth exon is 547 bp. These four exons are separated by introns that are 2006, 1407 and 637 bp in length, respectively. The exons encode a protein of 140 AA. The C-DOMAIN is encoded by EX1 and is 90 AA long. The C region also comprises the connecting region (CO) of 24 AA (encoded for 15 AA by EX2 and for nine AA by EX3) with a cysteine involved in the interchain disulfide binding, the transmembrane (TM) of 21 AA (encoded by the 3’ part of EX3) and the cytoplasmic region (CY) of five AA (encoded by the last part of EX3). The coding region of the Texel breed TRAC gene has 100 % nucleotide identity with the TRAC gene of the White alpine breed [7].

The deduced AA sequences of the sheep potential functional TRDV genes and in-frame pseudogenes were also manually aligned according to IMGT unique numbering for the V-REGION [27] (Fig. 2). Apart from the typical FR, the CDR and the conserved amino acids at the canonical positions, the TRDV1 subgroup genes share other characteristics including a 20 AA long highly conserved leader region (L-PART1 + L-PART2) and the YF motif at positions 102–103 located at the 3’ end of the FR3-IMGT (except for the TRDV1S8 pseudogene and the TRDV1S21 ORF). The TRDV1 CDR1-IMGT have a length of six, seven or nine AA. In contrast, the CDR2 are always three AA in length with the typical QXS motif (except for the TRDV1S18 gene). Interestingly, three genes, TRDV1S8, TRDV1S21 and TRDV1S24 contain a deletion that spans from the last FR2-IMGT AA to the sixth FR3-IMGT AA, resulting in the lack of the CDR2-IMGT. An identical deletion had already been found in the sheep TRDV1S29 gene [10]. The germline CDR3 are always four AA long except for the TRDV1S14 with two amino acids.

**Fig. 4:**
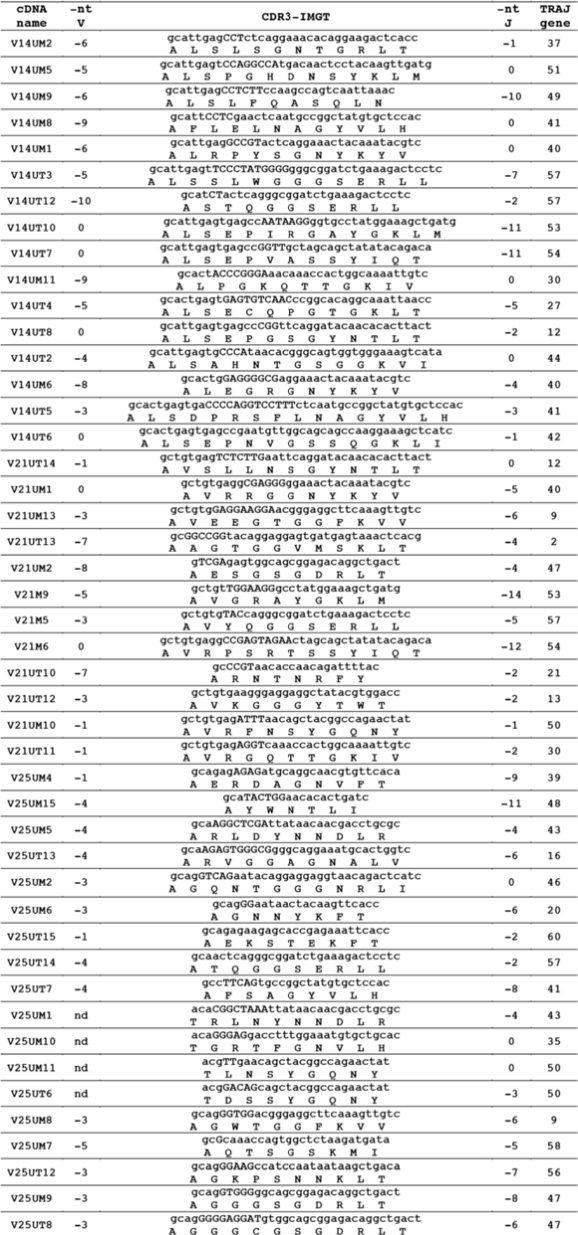
CDR3 nucleotide and predicted AA sequences retrieved from the TRA cDNA clones. CDR3-IMGT sequences are shown from codon 105 (codon after the 2nd-Cys 104 of the V-REGION) to codon 117 (codon before J-PHE 118 of the J-REGION) according to the IMGT unique numbering [27]. Nucleotides of the 3’V-REGION and of the 5’J-REGION are indicated in lower cases. Nucleotides that cannot be attributed to any V or J regions (N-nucleotides) are indicated in capital letters. Numbers in the left and right columns indicate the number of nt that are trimmed from the 3’V-REGION and 5’J-REGION, respectively. The name of the clones and the TRAJ genes is reported

When we compared the functional TRDV1 genes (16 sequences) identified in the Texel sheep assembly with the database sequence collections (40 sequences) available in the GEDI database [9, 10, 25], we found only six matched genes with a level of nucleotide identity ranging from 100 % to 97 % along the entire sequence (coding, intronic and flanking sequences). As a consequence, at least 50 functional germline TRDV1 genes might characterise the sheep TRA/TRD locus. Hence, considering also the pseudogenes and/or ORF we inferred that the sheep genome is characterised by the presence of about 58 diverse TRDV1 genes. These data are in agreement with that found in the bovine genome [14, 16].

The TRDV2, TRDV3 and TRDV4 subgroup genes all encode for a CDR1 that is seven AA in length, while the CDR2 is three AA long for TRDV2 and TRDV3 and six for TRDV4. Five and four AA are present in the germline CDR3 of the TRDV2 and TRDV3 and of the TRDV4 gene, respectively (Fig. 2).

The nucleotide and deduced AA sequences of the seven TRDD genes identified in the genome assembly are reported in Fig. 2. They consist of a nine (TRDD6), 11 (TRDD3 and TRDD7), 12 (TRDD1), 13 (TRDD4 and TRDD5) and 15 bp (TRDD2) sequence that can be productively read in its three coding frames. The 5’D-RS and 3’D-RS that flank the D-REGION are well conserved.

The TRDJ genes are typically 49–59 bp long (Fig. 2). Each TRDJ gene is flanked by the J-RS at the 5' end, and a donor splice at the 3' end. The coding regions were all predicted to be functional except for the TRDJ1-3 gene where the F-G-X-G motif whose presence characterises the functional J genes, is altered. This gene has never been identified within cDNA clones [8, 9]. The three functional TRDJ genes present 100 % nucleotide identity with the same genes identified in the sheep Altamurana breed [9].

The exon-intron organization of the TRDC gene was also determined (Fig. 2). The sheep TRDC gene encodes a protein of 155 AA. As already described [6], it is composed of three translated exons plus a fourth untranslated one. The C-DOMAIN is encoded by EX1 and is 93 AA long as in cattle. The C region also comprises the connecting region (CO) of 37 AA (encoded for 25 AA by EX2 and for 12 AA by EX3) with a cysteine involved in the interchain disulfide bridge, the transmembrane (TM) of 25 AA (encoded by the 3’ part of EX3) and the cytoplasmic region (CY) of five AA (encoded by the last part of EX3). Also in this case, no nucleotide substitutions were found in the coding region of the Texel breed TRDC gene with respect to the same gene of the Altamura breed [6].

### TRD chain transcript analysis as evidence for TRDD and TRDJ gene usage

To evaluate the contribution of each TRDD and TRDJ gene in the formation of the TRD chain repertoire, a total of 56 cDNA clones available in IMGT/LIGM-DB [29] and containing rearranged productive (no stop codons, in-frame junctions) V-D-J-C transcripts were analysed. 22 of these clones (AJ29 series) were derived from perinatal thymus of Altamurana breed, 26 (Z12 series) from peripheral blood of White Alpine breed and eight (AJ00 series) from pregnant uterus of Merino breed.

For a close inspection of the CDR3-IMGT, the nucleotide (nt) sequences from codon 105 (codon following the 2nd-CYS codon 104 of the V-REGION) to codon 117 (codon preceding the J-PHE 118 or J-TRP 118) that belongs to the F/W-G-X-G motif characteristic of the J-REGION (IMGT Repertoire, http://www.imgt.org, [27, 28]), were excised from each clone and reported in Fig. 3.

**Fig. 5:**
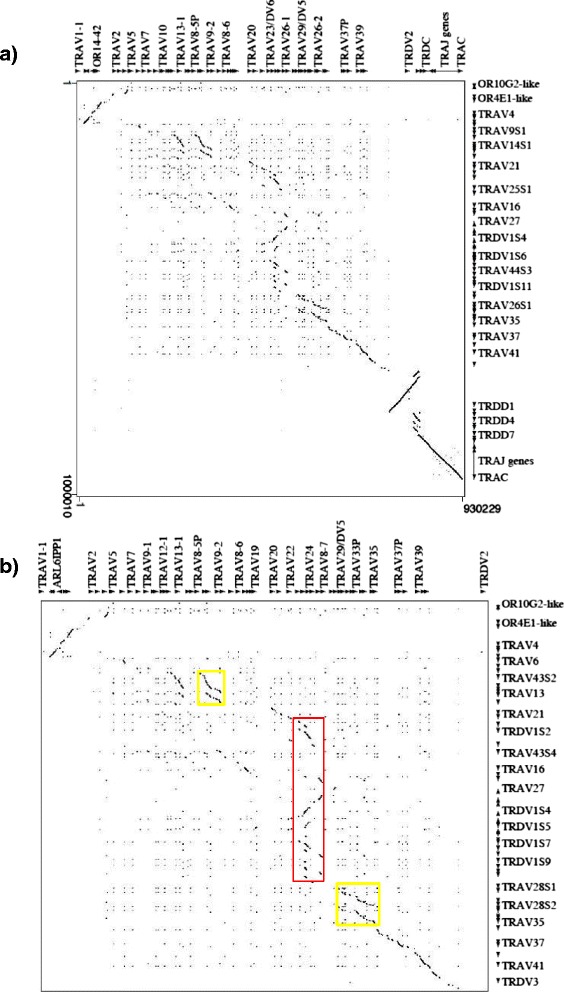
Dot-plot matrix of sheep/human TRA/TRD genomic comparison. Using the Pip-Maker program the entire sheep TRA/TRD locus (horizontal axis, positions 22731677 to 22831677 of the reference sequence NC_0194641) **(a)** or just the TR V region (horizontal axis, positions 22731677 to 22732377 to of the reference sequence NC_0194641) **(b)** have been plotted against the human counterparts (vertical axes, positions 21621904 to 22552132 in **(a)** and positions 21621904 to 21622706 in **(b)** of NC_000014). The transcriptional orientation of each gene is indicated by arrows and arrowheads. Coloured rectangles enclose TR V duplicated regions as referred in the text

By comparison with the TRDD genomic sequences, the nt located in each sequence were considered to belong to a TRDD gene if they constituted a stretch of at least five consecutive nt corresponding to one of the seven TRDD germline sequences. Thus, the sequences were grouped according to the absence or the presence of one or more TRDD genes. In particular, we observed six clones with no recognisable TRDD gene that could be interpreted as direct V-J junction. However, it is also possible that nucleotide trimming masked the initial participation of D gene during the rearrangement. In 13 clones, there is the presence of a single TRDD gene, while 21 and 15 clones have two or three TRDD genes, respectively. Only one clone (Z12989 in Fig. 3) contains four TRDD genes. All TRDD genes but one (TRDD3) were recovered within the cDNA clones. In some cases two or three TRDD genes are continuous, in others they are separated by P/N-nucleotides. As expected, the order of the genes, within the junction with more than one TRDD, corresponds to that found in the genome. A particular feature is the occurrence of nucleotide variations within the D region of four cDNAs (four clones) with respect to the germline sequences.

Although the number of clones are too low to be statistically significant we observed a preferential usage of the TRDD2 (25/50), TRDD5 (44/50) and TRDD7 (25/50) genes in the rearrangement. In contrast, the TRDD1 (10/50), TRDD4 (11/50) and TRDD6 (11/50) genes were used less frequently. In addition, a tissue-specific usage can be observed, with the TRDD2 (14/26) gene preferentially expressed in peripheral blood, the TRDD5 (7/8) in pregnant uterus and the TRDD7 (13/22) in thymus.

Inspection of the cDNA clones has allowed us to establish the contribution of the TRDJ genes (Fig. 3). A total of three TRDJ genes were recovered. The absence of the TRDJ3 gene confirms that it is a pseudogene. The TRDJ1 gene seems to be preferentially used in all the tissues (38/56). 14 out of 56 clones retain the TRDJ2 gene, while only four clones use the TRDJ4 gene.

By comparing with the TRDV and TRDJ germline sequences, the degree of trimming at the 3' end of the TRDV and at the 5' end of the TRDJ genes was estimated and reported in Fig. 3. The mean value of the trimming was 4.09 (range 0–13) for the 3' end of the TRDV and 2.12 (range 0–7) for the 5' end of the TRDJ genes.

The AA sequence of the V-delta CDR3 loop deduced from the nt sequence reveals that it is heterogeneous for AA composition and size. The mean length is 17.15 AA (range 8–26 AA) without difference in the three tissues. By comparison, the mean length of the sheep V-beta CDR3 is 13.18 AA long (range 9–20 AA) and is approximately the same in spleen, thymus and blood [30]. The V-delta CDR3 length seems to be correlated to the number of the TRDD genes incorporated in the loop, with a progression from clones without TRDD genes (mean 11.6 AA, range 8–14 AA) to those with one (mean 16.23 AA, range 13–20 AA), two (mean 18.0 AA, range 13–22 AA), or three TRDD genes (mean 20.6 AA, range 18–23 AA), reaching a maximum CDR3 length of 26 AA in the only clone with four TRDD genes.

Altogether these results suggest that a particularly long V-delta CDR3 may be a prerequisite to perform appropriate T cell functions and that this is achieved by increasing the number of TRDD genes used in the rearrangement rather than reducing the level of trimming at the ends of TRDV and TRDJ genes.

### Analysis of the expressed TRA repertoire

The published data on the sheep TRA chain repertoire are very limited if compared with those of the TRD, TRB and TRG chains. Only two entries are present in IMGT/LIGM-DB, one for a TRA mRNA [7] and one for a TRAV gene [6]. To evaluate features of the expressed repertoire from the analysis of the sheep TRA genomic repertoire identified in the genome assembly, we designed a panel of three TRAV subgroup-specific 5′ primers that we used in conjunction with a TRAC specific 3′ primer to amplify rearranged TRA chains from spleen and thymus RNA samples of a single sheep (see “Methods” section). We chose the TRAV14, TRAV21 and TRAV25 subgroups as representative of functional TRAV genes with four, three and two AA in the germline CDR3, respectively. A total of 48 different clones containing rearranged productive V-J-C transcripts were analysed. As expected, all of the TRAV genes belonged to the identified subgroups: 16 clones contain TRAV14 subgroup genes (series V14UM from spleen and series V14UT from thymus); 13 clones correspond to TRAV21 subgroup genes (series V21UM from spleen and series V21UT from thymus); 19 clones match with TRAV25 subgroup genes (series V25UM from spleen and series V25UT from thymus). The deduced AA sequences of the cDNA clones are reported in Additional file 7.

As the cDNA clones are derived from a single animal, we have compared the nucleotide sequences of the TRAV regions with each other to establish the level of gene and allelic nucleotide identity. Having found a minimum level of nucleotide identity of 98 % between two sequences within each TRAV subgroup, we have assumed that sequences with nucleotide identities ≥98 % represent allelic variants of genes; whilst, sequences with a level of identity <98 *%* correspond to distinct V genes.

Accordingly, we found three genes and one allelic variant for the TRAV14 as well as for the TRAV21 subgroups, and seven genes and one allelic variant for the TRAV25 subgroup, in contrast to a single gene for the TRAV21 and two for both the TRAV14 and the TRAV25 subgroups identified in the genomic assembly. Therefore, the majority of the expressed TRAV genes seem to be absent from the current assembly. Each gene was designated for simplicity with a letter and, if any, the alleles with an asterisk and a number (Additional file 7). When we compared the cDNA and the germline sequences of the TRAV genes, we found that only one of the expressed TRAV gene sequences (V21UT14) is identical to the TRAV21S1 gene. Whereas, the V25UT7 and V25UM4 clones show a percentage of identity of 99,07 and 98,46 respectively with respect to the germline TRAV25S2 and TRAV25S1 genes, indicating that they might represent allelic variants. The highest percentage of identity between cDNA and germline TRAV14 sequences was 97,64 %, suggesting that they could represent different genes or, alternatively, they might represent allelic variants of genes with a high level of polymorphisms between distinct sheep breeds (Gentile di Puglia and Texel).

A careful analysis of the AA composition of the genes and alleles reveals that differences are distributed along the entire sequence. The leader sequence (L-region) of TRAV25G gene is longer than the other TRAV genes by two AA (in italics in Additional file 7).

The expressed TRAJ genes corresponded to a genomic sequence with the exception of one AA change in the TRAJ41 (R117 > H) and TRAJ57 (D109 > G), however both AA changes are in the rearranged CDR3 (Additional file 7). 31 out of the functional TRAJ genes were identified, without any tissue-specific expression. Although the numbers are too low to be statistically relevant, a slight increase in the use of the TRAJ genes located in the TRAJ proximal region (TRAJ61 to TRAJ31) can be observed, with 35 clones containing TRAJ in this region.

For a close inspection of the V-alpha CDR3, the nt sequences from codon 105 to 117 were excised from each cDNA and analysed in detail (Fig. 4). The V-alpha CDR3 loop is heterogeneous for AA composition, especially due to the use of different TRAJ genes involved in the recombination process. The mean length is 11.08 AA (range 7–16 AA) and is approximately the same in spleen (mean 10.5 AA, range 7–13 AA) and thymus (mean 11.7 AA, range 8–16 AA). However, taking into account the TRAV genes, we found a slight increase of the V-alpha CDR3 length in the cDNA expressing TRAV14 genes (mean 12.9 AA, range 11–16 AA) compared to those expressing TRAV21 (mean 11.0 AA, range 8–13 AA) or TRAV25 (mean 9.6 AA, range 7–12 AA), which is probably related to the germline CDR3 lengths of four, three and two AA for TRAV14, TRAV21 and TRAV25, respectively.

By comparing with the TRAV and TRAJ germline sequences we were able to determine the degree of nt trimming of the 3'-V and 5'-J. The mean of nt deletion in 3'-V and 5'-J is 3.79 nt (range 0–10 nt) and 4.38 nt (range 0–12 nt), respectively, with no differences among the tissues. However, the degree of nt trimming is slightly higher for the TRAV14 genes (mean 4.75 nt, range 0–10) rather than for TRAV21 (mean 3.25 nt, range 0–8) and TRAV25 (mean 3.2 nt, range 1–5).

The trimming level at the 3'-V found for the TRAV14 genes, is similar to that reported for the TRDV genes. In both cases the size of the germline CDR3 is four. Therefore the germline gene structure might affect the characteristics of the rearranged CDR3.

### Comparison of sheep and human TRA/TRD loci

In order to better define the genomic structure of the sheep TRA/TRD locus, the determined sequence of the chromosome 7 genomic scaffold was aligned with the human counterpart, using the PipMaker program, and the alignment expressed as a dot-plot sequence comparison graph (Fig. 5). Inspection of the matrix highlights a high level of nt identity between sheep and human genes as indicated by dots and lines. Particularly, a prominent co-linearity between sheep and human sequences is shown at the 3’ end of the locus, as indicated by the long contiguous line that starts from the sheep TRAV28S1 and ends to the TRAC gene. Within this area, parallel lines show duplications around the sheep genomic regions encoding the seven TRDD genes; whereas, a perpendicular line indicates the presence of an inverted region which extends between the TRDV3 and the first TRDD gene.

A clear homology unit between sheep and human is also present at the 5' end of the locus although with an inverted orientation, as indicated by the orthogonal line (Fig. 5). Even if we cannot exclude the presence of inverted regions at the 5' and 3' end of the locus in the sheep genome with respect to human, the most likely hypothesis would be errors in the assembly.

A significant number of duplicative events are detected in the central region of the locus that has led to a substantial increase in the number of the sheep TR V genes. This is more evident in the enlarged version of the matrix (Fig. 5), corresponding to the only TR V containing regions, where parallel lines identify two duplication areas in which the sheep TRAV14 as well as the TRAV29 and TRAV26 gene subgroups have arisen through a tandem duplication event (yellow boxes). Furthermore, a noticeable feature is the presence of a section characterised by multiple homology regions, indicated by 11 short parallel lines or marked dots, due to the expansion of the sheep TRDV1 subgroup members (red box). The members of these expanded subgroups are intercalated with genes of sheep TRAV44 (four copies); TRAV22, TRAV25, TRAV42 and TRAV43 (two copies); TRAV16, TRAV23 and TRAV27 (one copy) subgroups. The section of homology corresponds to the human counterpart containing TRAV22-TRAV23/DV6-TRDV1-TRAV24-TRAV25-TRAV26-1 genes.

Apparently, due to the fragmentary nature of the assembly, the size of the blocks and the number of genes included in each block should be considered a minimum. However, the mechanism of TRDV1 amplification has undoubtedly facilitated the generation of multigene subgroups also among TRAV genes.

## Discussion

The locus TRA/TRD is the most complex among the TR loci. Its genomic organization is well known in “γδ low” species, such as human and mouse, however little is known in “γδ high” species, such as ruminants. Despite the numerous studies in cattle and the different genomic assemblies analysed, the extension and the gene content of the locus has not yet been ascertained with precision [13–16]. The accuracy of the results seems to be affected by the quality of the assembly within this complex locus as well as by the difficulty in the annotation of genes with a high degree of copy number variation (CNV) polymorphism.

In this paper, we have analysed the TRA/TRD locus in *Ovis aries* for the availability of the recent Oar_v3.1 release of the genomic assembly [18]. The scaffold of the chromosome 7 has been examined and a continuous region of about 1 Mb corresponding to the locus has been analysed (Fig. 1). The presence of two olfactory receptor genes at the 5’ end and the TRAC gene at the 3’ end, in conserved synteny with human and mouse, confirms that the sequence covers the entire extent of the locus. Moreover, additional TRAV and TRDV genes have been identified on 24 unplaced genomic scaffolds*,* which span about 280 kb and are expected to be located within the locus on chromosome 7 (Fig. 1, Table 1). The general genomic structure of the sheep TRA/TRD locus is confirmed to be as in the other mammalian species, with the TRDV intermingled with the TRAV genes at the 5’ of the locus, with seven TRDD, four TRDJ, one TRDC and one TRDV genes are nested between the TRA and TRD V genes and the 61 TRAJ genes preceding the TRAC gene.

A total of 66 TRAV and 28 TRDV genes have been identified. By the criterion that gene sequences having 75 % or greater of nucleotide identity belong to the same subgroup, the TRAV genes belong to 34 subgroups and the TRDV genes to four subgroups (Additional file 2).

The classification of the different sheep subgroups in comparison with human has allowed us to investigate the evolutionary relationships of the TRAV genes (Additional file 4). The clustering of the genes in the phylogenetic tree, shows 45 TRAV subgroups, with 30 of them being shared between sheep and humans. These subgroups can be classified as interspecies subgroups, indicating their occurrence as distinct subgroups prior to the divergence of the two mammalian species. Among these, the TRAV1 genes lie, in shared synteny at the beginning of the locus, interspersed between olfactory receptor genes in both species.

Eleven human TRAV subgroups are not represented in the sheep genome, while four TRAV subgroups (TRAV42 to TRAV45) have been found in sheep but not in human (Additional file 4). The paraphyletic groupings of these sheep-specific TRAV subgroups indicate that duplications of a shared ancestor gene followed by diversification and expansion events, have generated these species-specific genes.

Furthermore, the evolutionary relationship of the sheep and bovine TRAV genes was also investigated (data not shown; see “Methods” section). Although the incomplete nature of the sheep genomic assembly suggest that a substantial number of TRAV genes may not have been identified yet, six (TRAV7, TRAV15, TRAV30, TRAV31, TRAV32, TRAV40) out of 11 subgroups seem to be missing also in the bovine TRA/TRD locus (Additional file 8). It is to be noted that four of these six subgroups (TRAV15, TRAV30, TRAV31, TRAV32) have only pseudogene members in humans (Table 2). Moreover, the phylogenetic analysis reveals that the four sheep-specific TRA subgroups are also found in cattle (Additional file 8), indicating the occurrence of common ancestor genes in ruminants. Future information on the genomic organization of the TRA/TRD locus in other “γδ high” species will prove the origin of these new subgroups.

The provisional number of the TRAV genes identified in the sheep genomic assembly is further proven by our TRA chain expression data, carried out on a single animal (Additional file 7). In fact, the analysis of the repertoire regarding three different TRAV subgroups has revealed the presence of additional genes for each subgroup confirming the incompleteness of the genomic assembly sequence and that expansion events might have involved still other TRAV subgroups. However, CNV polymorphisms between different sheep breeds (Gentile di Puglia and Texel) have not to be excluded.

The multigene nature of several sheep TRAV subgroups explains the difference in the total amount of TRAV genes with respect to humans, although the number of the sheep subgroups is lower than in humans (Table 2). A possible explanation for the appearance in the sheep genome of these multigene subgroups could be in part the direct consequence to the gene expansion of the TRDV1 subgroup. The locus organisation of the sheep TRAV/TRDV genes appears to be consistent with this hypothesis since the members of the TRDV1 subgroup are always co-localised with TRAV genes, mostly belonging to expanded TRAV subgroups (Fig. 1, Table 1). Such concomitant expansion of TRAV and TRDV gene repertoires appears to be present also in the bovine [16] and pig [26] genome even if the partial sequencing of the pig TRA/TRD locus makes it less noticeable.

As regards to the sheep TRDV germline repertoire, previous evidence supporting the presence of one TRDV2 and one TRDV3 gene [25] as well as one TRDV4 gene located after the TRDC gene [10], are consistent with our findings (Fig. 1, Additional file 2). The sheep TRDV1 subgroup has been estimated to contain at least 40 genes [10], while only 25 TRDV1 genes have been identified in the genomic assembly. The existence of extensive gaps along the retrieved sequence, guarantees that additional TRDV1 genes may be still identified in the sheep genome assembly. Only complete assemblies of different genomes will fully elucidate the different V genes and alleles and solve such case as the TRDV1S12 gene contained in the unplaced scaffold NW_004080312 and the TRDV1S3 gene present in the Oar_v3.1 Chr7 genome sequence (Fig. 1, Table 1).

Although the 5’ part of the locus appears partial, the 260 kb in 3’ of the TRA/TRD sheep locus, extending from the TRDV3 to the TRAC gene, seems to be complete (Fig. 1). Seven TRDD genes were identified in 80 kb sequence. The same number of TRDD genes, covering about 60 kb, were identified in the pig genome with the third gene being a pseudogene [26]. Six TRDD genes were found in the bovine genome in a region of about 80 kb [16]. In contrast, there are only three TRDD genes on a 10.6 kb in humans and two TRDD genes on a 8.8 kb in mice.

All the sheep TRDD genes are potentially functional and show canonical recombination signal (5’D-RS and 3’D-RS) sequences (Fig. 2) even if the TRDD3 gene was not observed in the analysed TRD transcripts derived from published databases (Fig. 3), suggesting that, as in pig, it might be a pseudogene. The analysis of the same transcripts has demonstrated that a wide variety of CDR3 lengths, ranging from eight to 26 AA, is present, without any apparent bias towards a certain TRDV gene. The CDR3 size depends on the incorporation or not of different and sequential TRDD genes (up to four) in the formation of the TRD chain during the recombination process. In some cases, the recombination process has provided a perfect D-D fusion, in other cases, extra N-region nt have been added at the junction between two D genes. The end result is a CDR3 loop very variable in length as well as in AA composition. Similarly, the pig TRD chains can use up to four D genes [26], while in cattle, the TRD CDR3 shows combinations from one up to five TRDD genes with a range in length from nine to 37 AA [14]. Since the CDR3 composition and length influence the 3D configuration of the antigen-combining site [31], we speculate that V-delta domain with a long CDR3 loop have increased flexibility and are therefore better suited to recognise a large number of antigenic conformations. It is interesting how the VH CDR3 loop in cattle antibodies also shows extensive heterogeneity in size, ranging from three even to 61 codons. However, based on the genomic characterisation of the bovine IGHD genes, it has been demonstrated that a direct contribution of the germline IGHD2 gene rather than D-D fusions explains the origin of the long CDR3. In fact, the IGHD2 represents the single longest IGHD gene among ten identified in the cattle genome, potentially capable of directly encoding 49 codons. Further, specific insertion of novel ‘conserved short nucleotide sequences’ at the IGHV–IGHD junction provides the molecular basis of the origin of exceptionally long VH CDR3 [32].

A preferential usage of the TRDD and TRDJ genes, proceeding from 5 ' to 3' in the locus was also observed (Fig. 3), but further expression studies will be needed to confirm that the genomic organization of the genes might affect the characteristics of the rearranged CDR3.

Similarly, our TRA chain expression analysis has revealed a slight increase in the use of the TRAJ genes located in the proximal region (Fig. 4), consistent with the widely accepted view that TRAV-TRAJ recombination proceeds in a coordinated, sequential manner from proximal to progressively more distal TRAV and TRAJ genes [33, 34].

## Conclusion

The present study identifies the genomic structure and the gene content of the TRA/TRD locus in the *Ovis aries* whole genome assembly. Overall, the sheep genomic organization is highly conserved with respect to the other mammalian species, with the TRD genes nested within the TRA locus. Despite the fragmented and incomplete nature of the current genome assembly in the 5' part of the locus, through phylogenetic and expression analyses, we provide evidence of an increase in the total amount of the sheep TRAV genes compared to those of humans due to the genomic expansion of a consistent number of different subgroups. A substantial proportion of these expanded TRAV genes may have occurred as consequence of the amplification of the TRDV1 subgroup genes, typically associated with the “γδ high” species.

The essential completeness of the assembly of the 3' part of the locus has allowed a correct interpretation of the CDR3 formation for the TRA and TRD chain repertoires. If the mechanism for generating diversity in sheep TRA chain appears to adhere to the paradigms established through the study of human and mouse (no substantial difference in the mechanism of the recombination process for the TRA chain between sheep and human was observed), the use of up to four out of seven TRDD genes, and the possibility of the addition of N nucleotides on either side of these, makes the potential sheep TRD chain much larger than any known TR chain repertoire. This situation is quite different from humans, and suggests that the differences between “γδ high” and “γδ low” species in distribution, diversity, and function of γδ T cells may be substantial.

## Methods

### Genome and sequence analyses

To determine TRA/TRD locus location, the *Ovis aries* breed Texel Oar_v3.1 whole genome shotgun sequence was searched using the BLAST algorithm. A sequence of 1 Mb (gaps included) was retrieved directly from the chromosome 7 reference sequence NC_019464.1 available at NCBI from positions 22731677 to 22831677. Additional 24 unplaced genomic scaffolds (NW_004080312, NW_004080344, NW_004080351, NW_004080352, NW_004080507, NW_004080664, NW_004080708, NW_004080772, NW_004080962, NW_004081110, NW_004081259, NW_004081534, NW_004081794, NW_004081954, NW_004082010, NW_004082156, NW_004082203, NW_004082507, NW_004082570, NW_004082847, NW_004083226, NW_004083566, NW_004084646 and NW_004084647), containing TRAV and TRDV genes, were also recovered.

The TRA and TRD genes were identified using the human and sheep sequences available in the GEDI (for GenBank/ENA/DDBJ/IMGT/LIGM-DB) databases. Locations of the TRA and TRD genes are provided in Additional file 2.

Computational analysis of the sheep TRA/TRD locus was conducted with the following programs: RepeatMasker for the identification of the genome-wide repeats and low complexity regions (Smit, A.F.A., Hubley R., Green P., RepeatMasker at http://www.repeatmasker.org) and Pipmaker (http://pipmaker.bx.psu.edu/pipmaker/, [19]) for the alignment of the determined sheep sequence in NC_019464.1 with the human counterpart (NC_000014: positions 21621904–22552132)

### Classification of the TRA and TRD genes

Sheep TRA and TRD genes were named following the IMGT nomenclature established for human and mouse (IMGT®, http://www.imgt.org, [21]): TR V genes were assigned to different subgroups on the basis of the percentage of nt identity by using Clustal W2 alignment tool available at EMBL-EBI website (http://www.ebi.ac.uk/).

### Phylogenetic analyses were performed in order to classify the sheep TRAV and TRDV subgroups.

Due to the fragmented and incomplete nature of the genomic assembly, a temporary designation is used for multigene subgroups in which the arabic number (for the subgroup) is followed by the letter S, themselves followed by the number of the gene in the subgroup. For the TRAV genes, we used the nt sequences of the V-REGION of TRAV genes from sheep and human, whereas, the entire nt coding regions (L-PART1 + V-EXON) were used for the sheep TRDV analysis. The human TRAV gene sequences were retrieved from IMGT® (IMGT Repertoire, http://www.imgt.org, IMGT/GENE-DB, [4]).

Multiple alignments of the sequences under analysis were carried out with the MUSCLE program [35]. Evolutionary analyses were conducted in MEGA5 [36]. We used the neighbor-joining (NJ) method to reconstruct the phylogenetic trees [22]. The evolutionary distances were computed using the p-distance method [37] and are in the units of the number of base differences per site. The analysis involved 109 nucleotide sequences. All positions containing gaps and missing data were eliminated.

A phylogenetic analysis was also performed, as described above, combining, in the same alignment, the V-REGION nucleotide sequences of the sheep TRAV genes with those of the bovine TRAV genes deduced by Connelley et al. [16].

TRDD, TRDJ, TRDC, TRAJ and TRAC were named according to their location from 5′ to 3′ in the locus. As a consequence, the previous TRDJ classification [9] has been updated.

### cDNA cloning and analysis

Total RNA was extracted from the spleen and thymus of a 100 days old animal of Gentile di Puglia breed reared in our geographic area, using the Trizol method according to the manufacturer’s instructions (Life Technologies). One RNA sample from each tissue was obtained. About 1 μg of total RNA was converted to cDNA using the Superscript III reverse transcriptase (Life Technologies) with priming by the oligo(dT)15 primer.

Six different PCRs were then conducted, three for each RNA sample, using TRAV14 (V14U: 5’-GCAGCACATACCCAGCAA-3’), TRAV21 (V21U: 5’-AGCCTCTTTTATCCTGTG-3’) and TRAV25 (V25U: 5’-AGGAGCAGGCAGATTCAG-3’) subgroup-specific 5′ primer in combination with a TRAC (SP3 5’-ACGGTGCTGTCTGTTTTGTG-3’) specific 3′ primer.

Individual reactions were composed of 10 μl of the purified cDNA, 10 pmol each of the relevant 5′ and 3′ primers, 0.5 units of Platinum Taq Polymerase (Life Technologies) and 5 μl 10× buffer, 2 μl MgCl2 (50 mM), 1 μl dNTP (10 mM) per 50 μl reaction. Cycling conditions were 5 min. at 95 °C, 35 cycles of (30s at 95 °C, 45 s. at 55-58 °C, 1 min. at 72 °C), and a final extension period of 30 min. at 72 °C.

The PCR products were then purified with High Pure PCR Product Purification Kit (Roche Diagnostic GmbH) and cloned into pCR-XL-TOPO vector (TOPO XL PCR cloning Kit, Life Technologies). 15 random selected positive clones for each cloning were sequenced by a commercial service (BMR-Genomics).

cDNA sequence data were processed and analyzed using the Blast program (http://blast.ncbi.nlm.nih.gov/Blast.cgi), Clustal W2 (http://www.ebi.ac.uk/Tools/msa/clustalw2/) and IMGT® tools [IMGT/V-QUEST [23, 24] with integrated IMGT/JunctionAnalysis tools [38, 39] and the IMGT unique numbering for V domain (IMGT®, http://www.imgt.org, [27]) 48 TRA productive (no stop codons, in-frame junction) transcripts have been obtained, with an identical cDNA shared between spleen and thymus (V11UM1/T3). All cDNA sequences were registered in ENA for GEDI public availability under the Accession numbers from LN846380 to LN846426.

## Additional files

Additional file 1:
**Analysis of the genomic structure of the sheep TRA/TRD locus.** The sheep masked sequence was aligned versus itself and the alignment is showed as percentage identity plot (pip). The position and orientation of all genes are indicated, together with the location and orientation of the interspersed repeats as well. Horizontal lines represent ungapped alignments at the percentage identity corresponding to the scale on the right to the sequences. The presence in the pip of superimposed lines indicates the occurrence of redundant matches along the entire region. The clearest matches are in corresponding of all sheep variable genes due to the homology among genes. (PPT 570 kb) Additional file 2:
**Description of the TRA/TRD genes in the sheep genome assembly.** The position of all genes and their classification and functionality are reported. (ZIP 39.6 kb) Additional file 3:
**Description of the TRAV/TRDV ORF and pseudogenes.** (DOC 87 kb) Additional file 4:
**The NJ tree inferred from the sheep and human TRAV gene sequences.** The evolutionary analysis was conducted in MEGA5 [36]. The percentage of replicate trees in which the associated taxa clustered together in the bootstrap test (1000 replicates) is shown next to the branches [40]. The trees are drawn to scale, with branch lengths in the same units as those of the evolutionary distances used to infer the phylogenetic trees. The evolutionary distances were computed using the p-distance method [37] and are in the units of the number of base differences per site. The sheep TRAV subgroup classification is according to the corresponding human TRAV subgroups. Sheep-specific subgroups are numbered from TRAV42 (boxed in the right column). The TRAV genes found in humans and not in sheep are boxed in the tree. The gene functionality according to IMGT rules (F: functional, ORF: open reading frame, P: pseudogene) is indicated. For each subgroup, the CDR1-IMGT, CDR2-IMGT and CDR3-IMGT lengths are reported between brackets in the right column: when different between species, the first is for the human subgroup and the second is for the sheep subgroup. For TRAV42 to TRAV45, the CDR-IMGT lengths are for the sheep subgroup only (no corresponding subgroup in humans). The IMGT 6-letter standardised abbreviation for taxon is used: three letters for genus and three letters for species (*Homsap*, *Oviari*). (PPT 7.56 kb) Additional file 5:
**Evolutionary relationship of the sheep TRDV genes.** The TRDV nomenclature is according to IMGT-ONTOLOGY concepts of classification [28]. (PDF 7.56 kb) Additional file 6:
**TRA genes.** In a, the IMGT Protein display of the sheep TRAV genes. Only functional genes ORF and in-frame pseudogenes are shown. The description of the strands and loops and of the FR-IMGT and CDR-IMGT is according to the IMGT unique numbering for V-REGION [27]. The CDR-IMGT AA lengths are indicated in square brackets. nd: not defined (indicates that the AA sequence of the TRAV45S1 gene is incomplete and its functionality cannot be defined). In b, nucleotide and deduced AA sequences of the sheep TRAJ genes. The numbering adopted for the gene classification is reported on the left of each gene. The functionality is also reported. The consensus sequences of the J-heptamer and J-nonamer [41] are provided at the top of the figure and underlined. The donor splice site for each TRAJ is also shown. The canonical F/W-G-X-G amino acid motifs are underlined. In c, IMGT Protein display of the TRAC gene. Description of the strands and loops is according to the IMGT unique numbering for C-DOMAIN [42]. (ZIP 325 kb) Additional file 7:
**Protein display of the TRA cDNA clones.** The TRAV and TRAJ genes, named in accordance with the criteria specified in the text, are listed respectively at the left and the right of the figure. The cDNA clones are grouped by TRAV subgroups and those containing the same TRAV gene and allele were boxed and classified with alphabetic letters. Leader region (L-PART1 + L-PART2), CDR-IMGT and FR-IMGT are also indicated, according to the IMGT unique numbering for V-DOMAIN [27]. The five conserved AA of the V-DOMAIN (1st-CYS 23, CONSERVED-TRP 41, hydrophobic AA 89, 2nd-CYS 104 and J-PHE 118) are indicated in bold. The AA changes between genes and allele of the same TRAV subgroup, if any, are shaded. The name of the clones is also reported. (DOC 135 kb) Additional file 8:
**Corrispondence between ovine and bovine germline TRAV repertoires.** The bovine TRAV gene sequences were retrieved by Connelley et al. [16]. Correspondence was established as reported in Methods. (DOC 59 kb)
