# Supplementary material for: Sheep (Ovis aries) T cell receptor alpha (TRA) and delta (TRD) genes and genomic organization of the TRA/TRD locus
Source: BMC Genomics. 2015 Sep 18;16:709. doi: 10.1186/s12864-015-1790-z (PMC4574546; doi:10.1186/s12864-015-1790-z)

## Slide 1
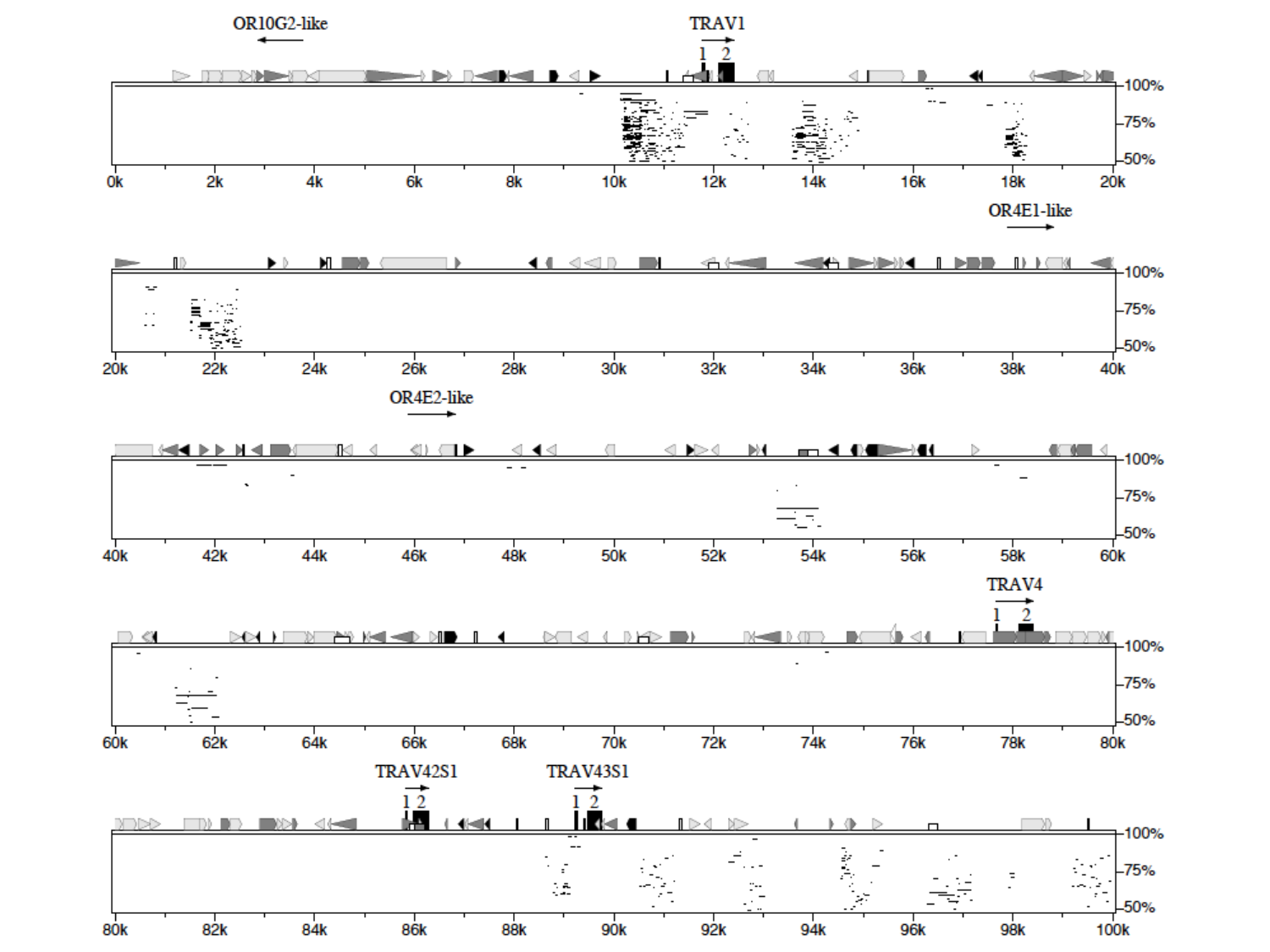

## Slide 2
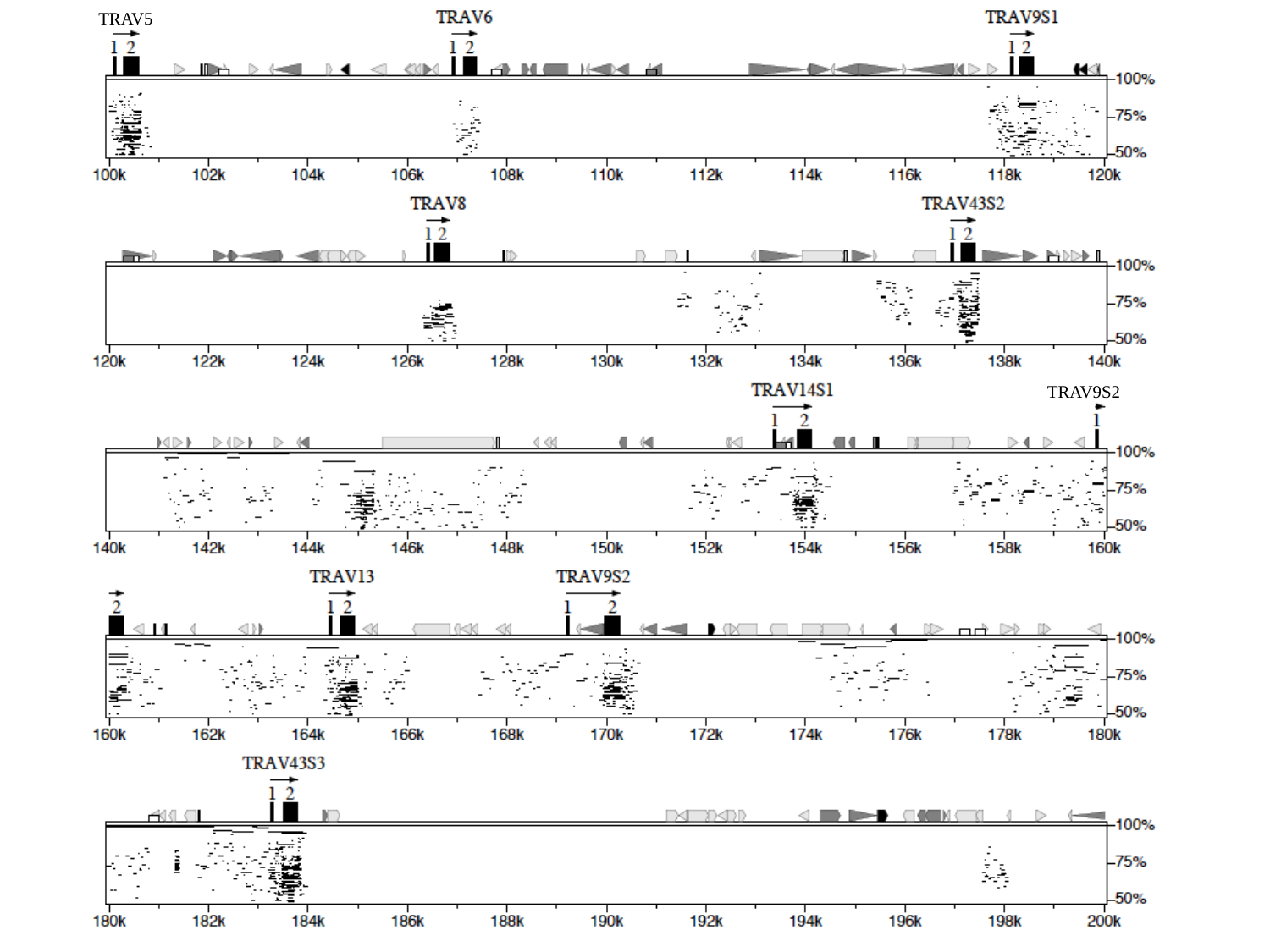

TRAV5
TRAV9S2

## Slide 3
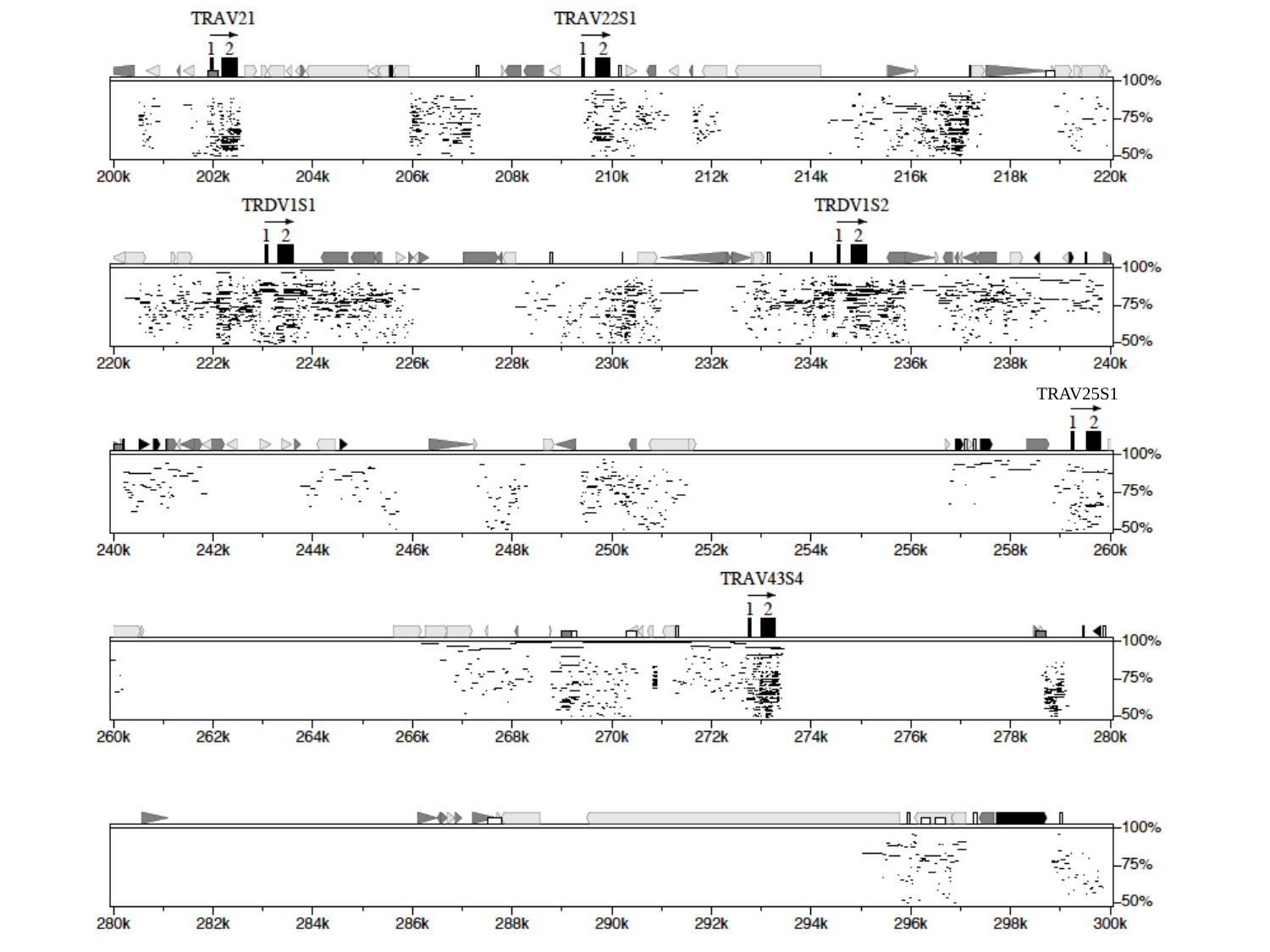

TRAV25S1

## Slide 4
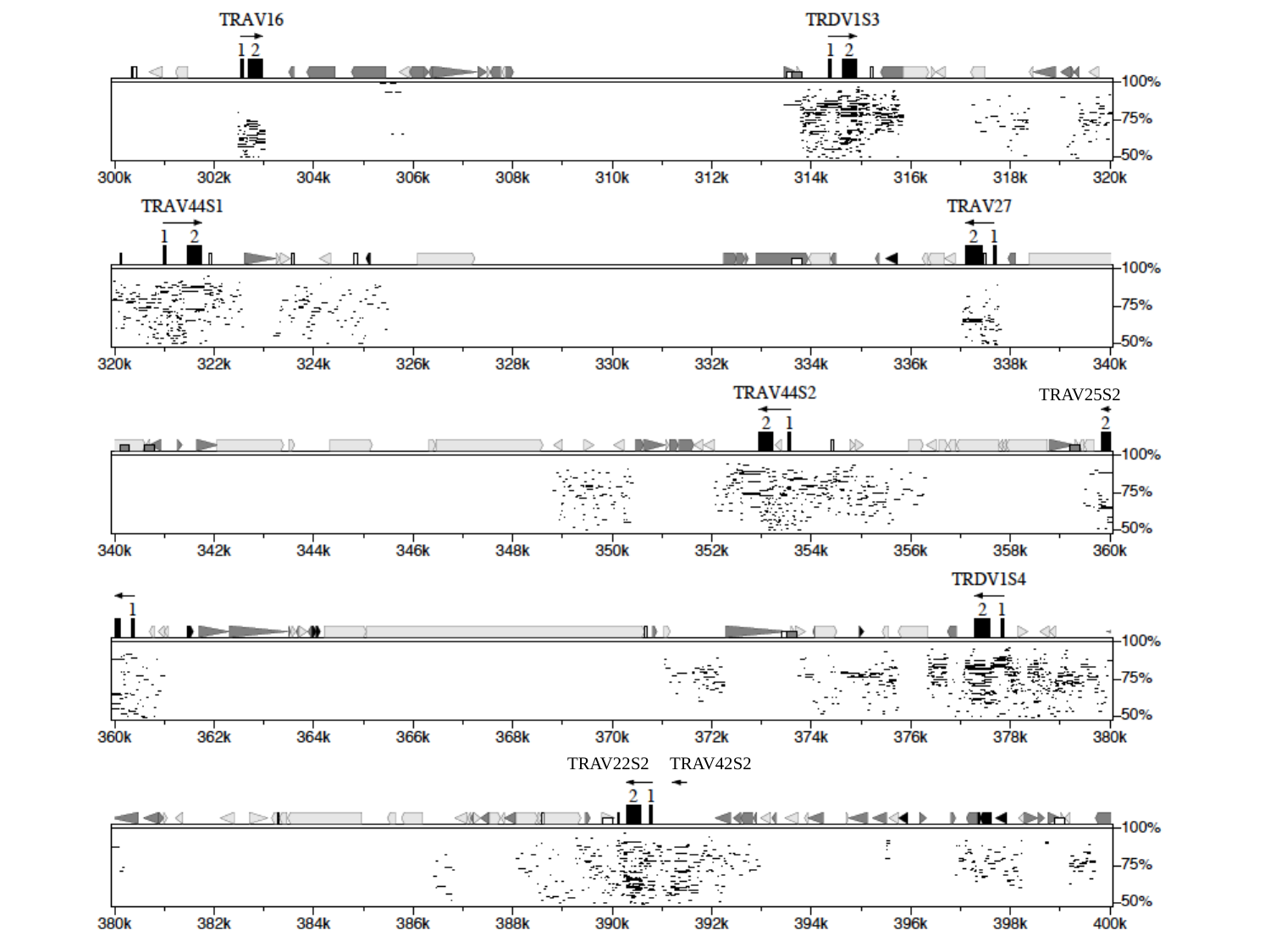

TRAV25S2
TRAV22S2
TRAV42S2

## Slide 5
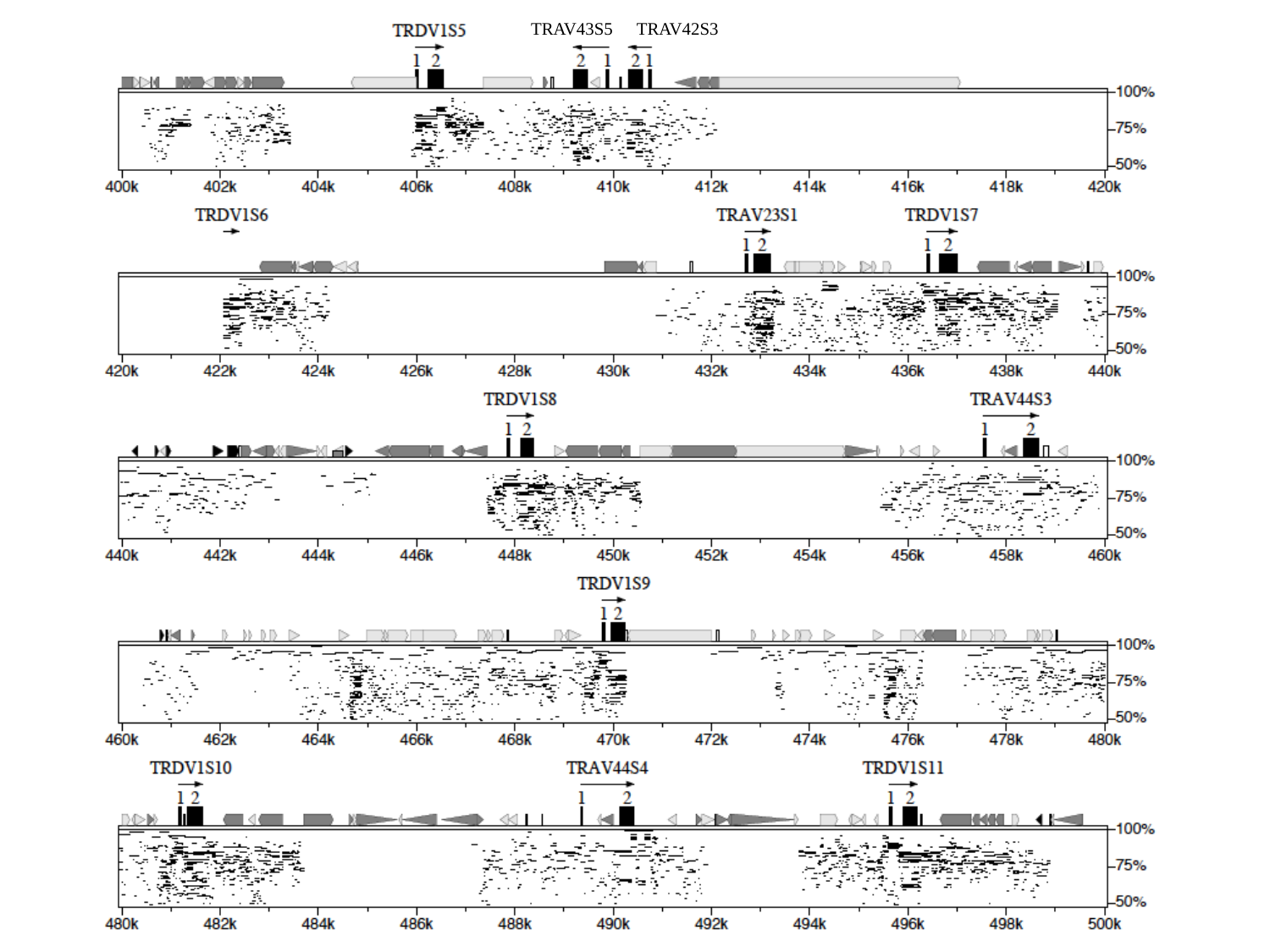

TRAV43S5
TRAV42S3

## Slide 6
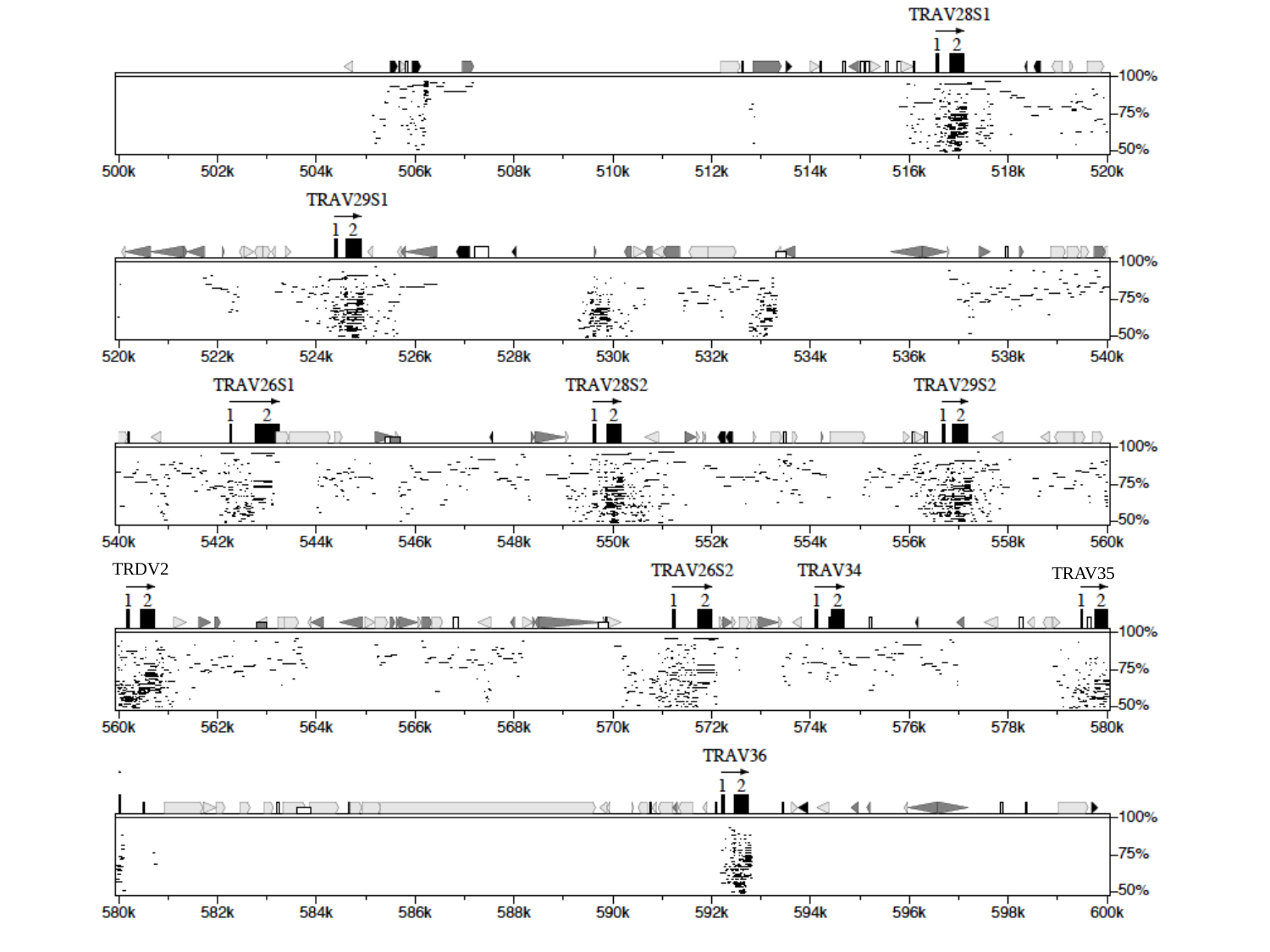

TRDV2
TRAV35

## Slide 7
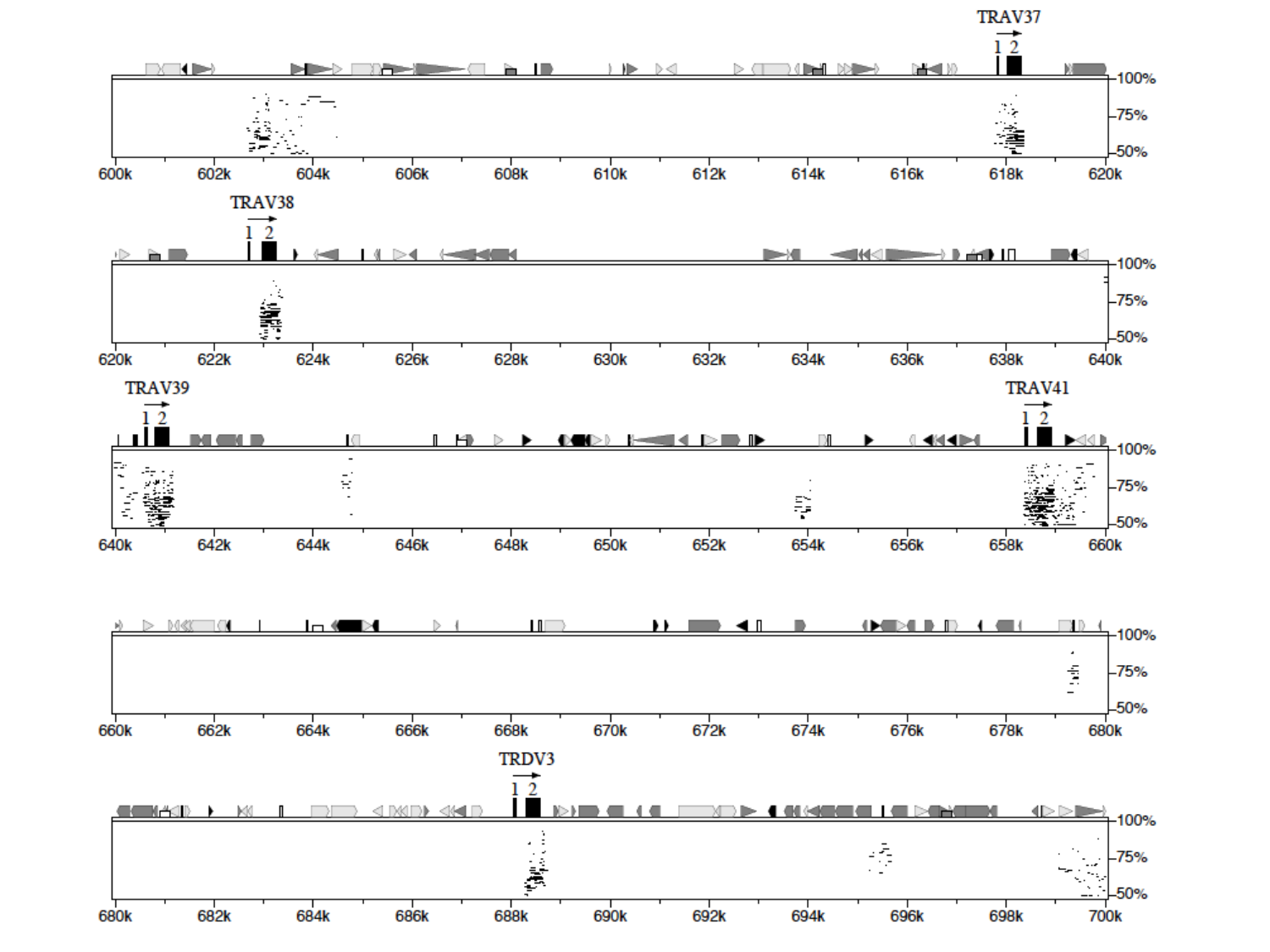

## Slide 8
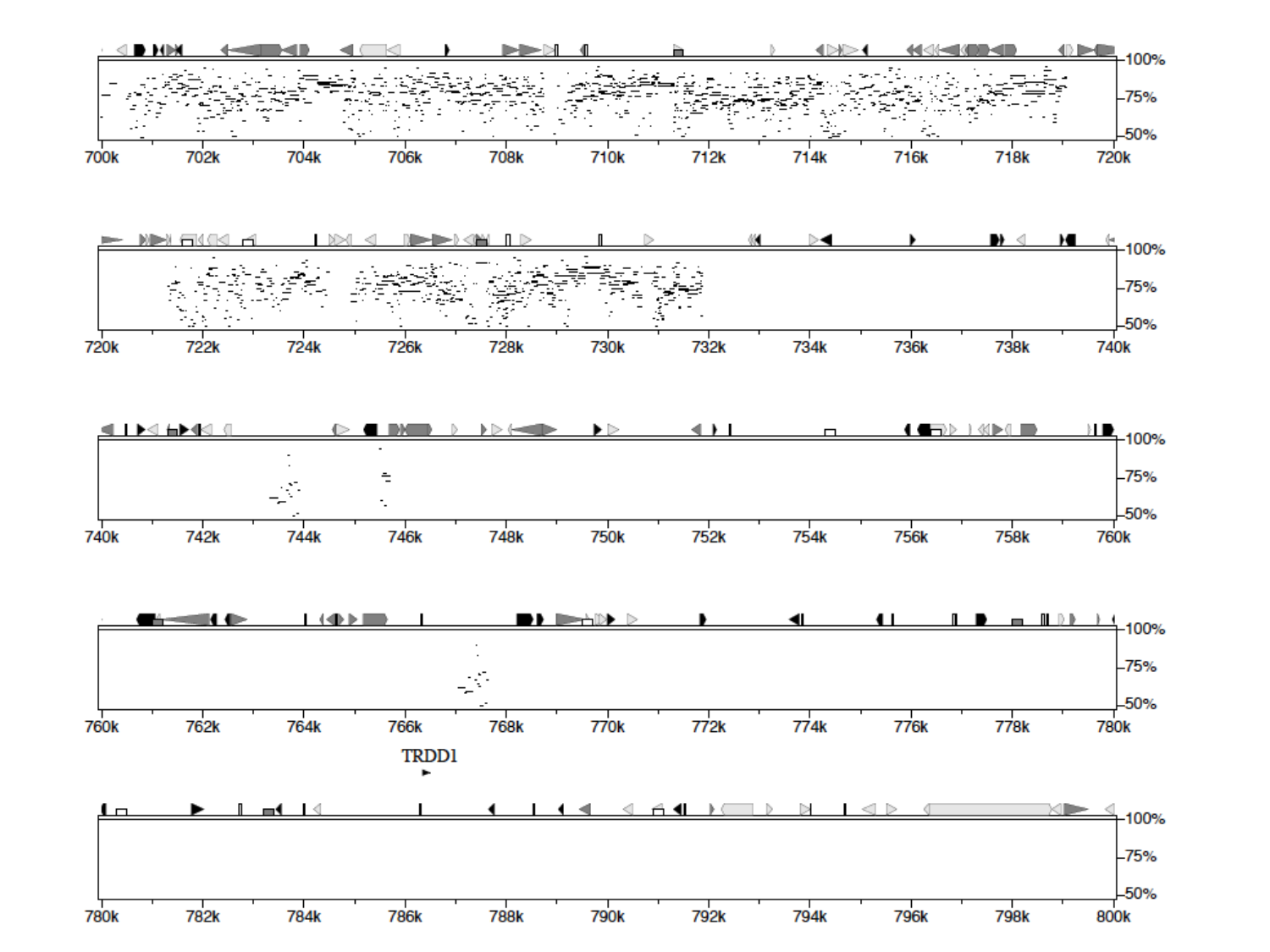

## Slide 9
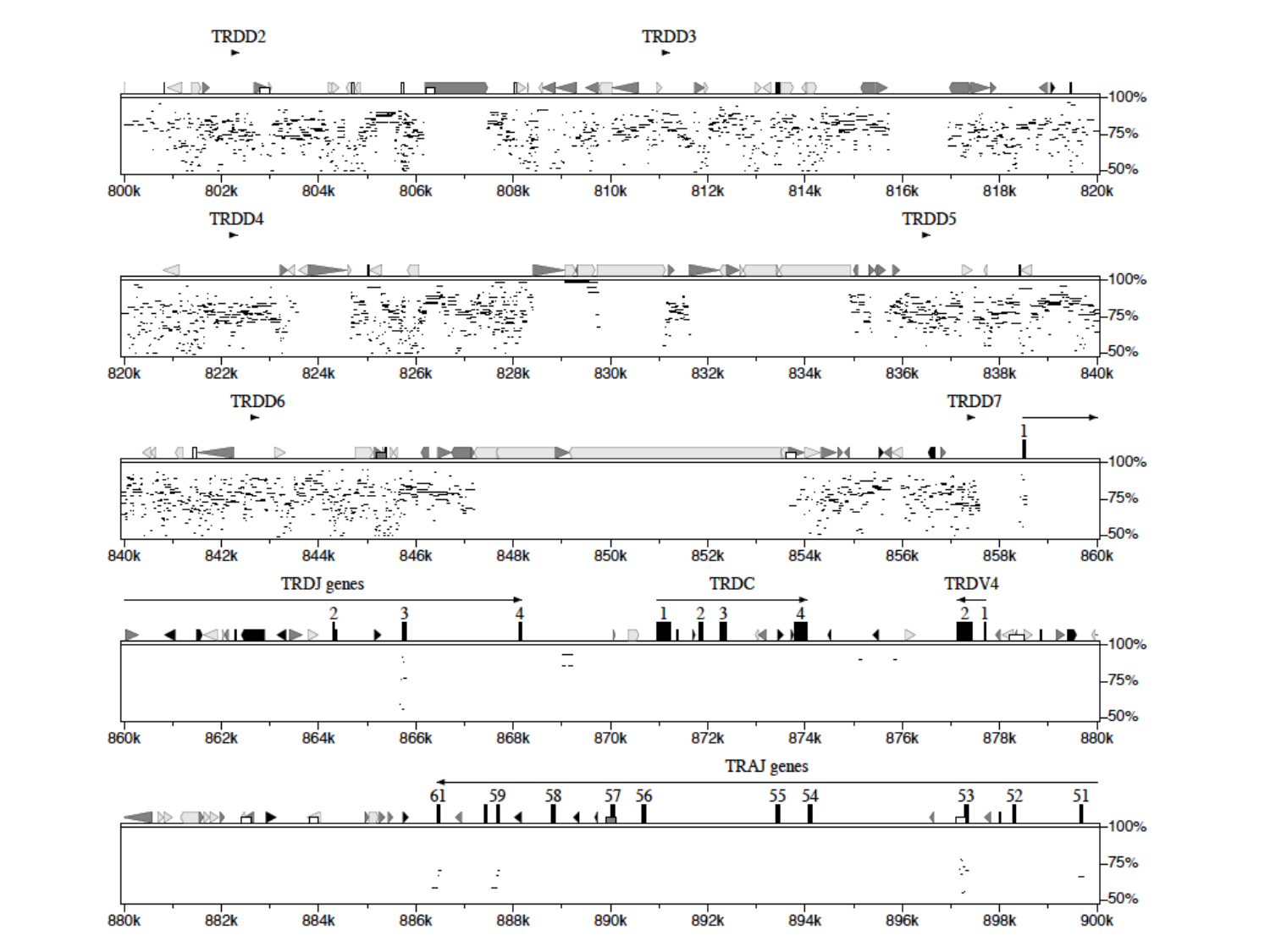

## Slide 10
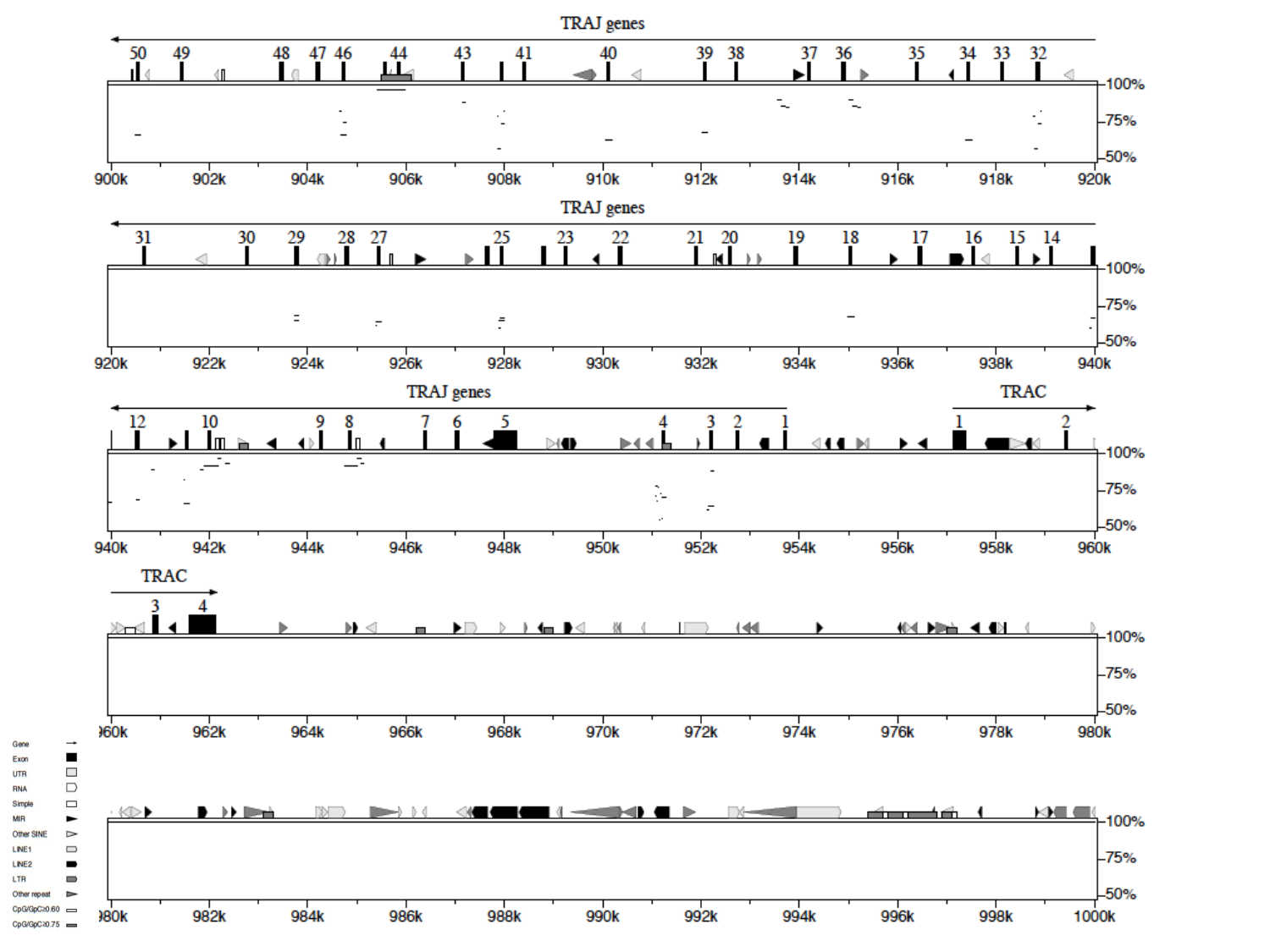

Supplement: Additional file 1: — Analysis of the genomic structure of the sheep TRA/TRD locus. The sheep masked sequence was aligned versus itself and the alignment is showed as percentage identity plot (pip). The position and orientation of all genes are indicated, together with the location and orientation of the interspersed repeats as well. Horizontal lines represent ungapped alignments at the percentage identity corresponding to the scale on the right to the sequences. The presence in the pip of superimposed lines indicates the occurrence of redundant matches along the entire region. The clearest matches are in corresponding of all sheep variable genes due to the homology among genes. (PPT 570 kb) [file 12864_2015_1790_MOESM1_ESM.ppt]
