# Supplementary material for: Sheep (Ovis aries) T cell receptor alpha (TRA) and delta (TRD) genes and genomic organization of the TRA/TRD locus
Source: BMC Genomics. 2015 Sep 18;16:709. doi: 10.1186/s12864-015-1790-z (PMC4574546; doi:10.1186/s12864-015-1790-z)
Supplement: Additional file 2: — Description of the TRA/TRD genes in the sheep genome assembly. The position of all genes and their classification and functionality are reported. (ZIP 39.6 kb) [file 12864_2015_1790_MOESM2_ESM.doc]

**Additional file 2. Description of the TRA/TRD genes in the sheep genome assembly**. The position of all genes and their classification and functionality are reported.

| **Gene classification** | **Functionality** | **Scaffold number** | **NCBI Reference Sequence** | **Position**^a^ |
| --- | --- | --- | --- | --- |
| TRAV1 | F | Chr7 | NC_019464 | 22819910-22819282 |
| TRAV2 | ORF | ChrUn.7048 | NW_004084647 | 1150-1632 |
| TRAV3 | F | ChrUn.7047 | NW_004084646 | 258-690 |
| TRAV4 | F | Chr7 | NC_019464 | 22516763-22517316 |
| TRAV5 | F | Chr7 | NC_019464 | 22731088-22731599 |
| TRAV6 | P | Chr7 | NC_019464 | 22724283-22724787 |
| TRAV8 | P | Chr7 | NC_019464 | 22704834-22705284 |
| TRAV9S1 | P | Chr7 | NC_019464 | 22713086-22713555 |
| TRAV9S2 | F | Chr7 | NC_019464 | 22671379-22671843 |
| TRAV9S3 | F | ChrUn.3065 | NW_004080664 | 1216-1677 |
| TRAV9S4 | F | ChrUn.4971 | NW_004082570 | 848-1309 |
| TRAV10 | F | ChrUn.4195 | NW_004081794 | 10825-11397 |
| TRAV12 | F | ChrUn.3363 | NW_004080962 | 3603-4156 |
| TRAV13 | F | Chr7 | NC_019464 | 22666738-22667245 |
| TRAV14S1 | ORF | Chr7 | NC_019464 | 22677552-22678317 |
| TRAV14S2 | F | Chr7 | NC_019464 | 22661407-22661700 |
| TRAV16 | F | Chr7 | NC_019464 | 22528703-22529146 |
| TRAV17 | F | ChrUn.2908 | NW_004080507 | 6350-7085 |
| TRAV18S1 | F | ChrUn.2745 | NW_004080344 | 2225-2744 |
| TRAV18S2 | F | ChrUn.3935 | NW_004081534 | 5283-5791 |
| TRAV21 | F | Chr7 | NC_019464 | 22629733-22629199 |
| TRAV22S1 | P | Chr7 | NC_019464 | 22622283-22621708 |
| TRAV22S2 | F | Chr7 | NC_019464 | 22440867-22441394 |
| TRAV22S3 | F | ChrUn.2752 | NW_004080351 | 1533-2056 |
| TRAV22S4 | P | ChrUn.3109 | NW_004080708 | 71-736 |
| TRAV22S5 | ORF | ChrUn.3511 | NW_004081110 | 4965-5488 |
| TRAV22S6 | F | ChrUn.3511 | NW_004081110 | 22143-22710 |
| TRAV23S1 | P | Chr7 | NC_019464 | 22398485-22398987 |
| TRAV23S2 | P | ChrUn.4908 | NW_004082507 | 1243-1750 |
| TRAV25S1 | P | Chr7 | NC_019464 | 22572446-22571863 |
| TRAV25S2 | F | Chr7 | NC_019464 | 22471277-22471850 |
| TRAV26S1 | F | Chr7 | NC_019464 | 22288633-22289429 |
| TRAV26S2 | F | Chr7 | NC_019464 | 22259663-22260462 |
| TRAV27 | F | Chr7 | NC_019464 | 22494021-22494569 |
| TRAV28S1 | F | Chr7 | NC_019464 | 22314572-22315132 |
| TRAV28S2 | F | Chr7 | NC_019464 | 22281521-22282074 |
| TRAV29S1 | F | Chr7 | NC_019464 | 22306774-22307301 |
| TRAV29S2 | F | Chr7 | NC_019464 | 22274484-22275013 |
| TRAV34 | P | Chr7 | NC_019464 | 22256999-22257572 |
| TRAV35 | P | Chr7 | NC_019464 | 22251625-22252211 |
| TRAV36 | F | Chr7 | NC_019464 | 22238932-22239477 |
| TRAV37 | P | Chr7 | NC_019464 | 22213375-22213857 |
| TRAV38 | F | Chr7 | NC_019464 | 22208412-22208994 |
| TRAV39 | ORF | Chr7 | NC_019464 | 22190580-22191071 |
| TRAV41 | F | Chr7 | NC_019464 | 22172766-22173304 |
| TRAV42S1 | ORF | Chr7 | NC_019464 | 22745397-22745863 |
| TRAV42S2 | P | Chr7 | NC_019464 | 22440175-22440455 |
| TRAV42S3 | P | Chr7 | NC_019464 | 22420909-22421356 |
| TRAV42S4 | F | ChrUn.2713 | NW_004080312 | 18959-19431 |
| TRAV43S1 | F | Chr7 | NC_019464 | 22741929-22742458 |
| TRAV43S2 | P | Chr7 | NC_019464 | 22694266-22694742 |
| TRAV43S3 | F | Chr7 | NC_019464 | 22647898-22648425 |
| TRAV43S4 | F | Chr7 | NC_019464 | 22558929-22558400 |
| TRAV43S5 | P | Chr7 | NC_019464 | 22421764-22422473 |
| TRAV44S1 | ORF | Chr7 | NC_019464 | 22509923-22510691 |
| TRAV44S2 | F | Chr7 | NC_019464 | 22478091-22479006 |
| TRAV44S3 | F | Chr7 | NC_019464 | 22373028-22374128 |
| TRAV44S4 | P | Chr7 | NC_019464 | 22341247-22342333 |
| TRAV44S5 | F | ChrUn.2713 | NW_004080312 | 9166-9939 |
| TRAV44S6 | F | ChrUn.4908 | NW_004082507 | 24215-24983 |
| TRAV44S7 | P | ChrUn.4908 | NW_004082507 | 45289-46383 |
| TRAV45S1 | nd^b^ | ChrUn.3173 | NW_004080772 | 1-189 |
| TRAV45S2 | F | ChrUn.3173 | NW_004080772 | 12992-13460 |
| TRAV45S3 | F | ChrUn.3935 | NW_004081534 | 9337-9816 |
| TRAV45S4 | F | ChrUn.3935 | NW_004081534 | 20286-20766 |
| TRAV45S5 | P | ChrUn.5248 | NW_004082847 | 2915-3210 |
| TRDV1S1 | F | Chr7 | NC_019464 | 22608631-22608065 |
| TRDV1S2 | F | Chr7 | NC_019464 | 22597146-22596576 |
| TRDV1S4 | P | Chr7 | NC_019464 | 22453820-22454388 |
| TRDV1S5 | F | Chr7 | NC_019464 | 22425693-22425133 |
| TRDV1S6 | nd^b^ | Chr7 | NC_019464 | 22409591-22409288 |
| TRDV1S7 | P | Chr7 | NC_019464 | 22395292-22394686 |
| TRDV1S8 | P | Chr7 | NC_019464 | 22383829-22383293 |
| TRDV1S9 | P | Chr7 | NC_019464 | 22361886-22361434 |
| TRDV1S10 | P | Chr7 | NC_019464 | 22350504-22350033 |
| TRDV1S11 | F | Chr7 | NC_019464 | 22336042-22335479 |
| TRDV1S12 | F | ChrUn.2713 | NW_004080312 | 1398-1952 |
| TRDV1S13 | F | ChrUn.2753 | NW_004080352 | 1952-2528 |
| TRDV1S14 | F | ChrUn.3511 | NW_004081110 | 10550-11115 |
| TRDV1S15 | P | ChrUn.3660 | NW_004081259 | 7509-8071 |
| TRDV1S16 | P | ChrUn.4355 | NW_004081954 | 2741-3251 |
| TRDV1S17 | F | ChrUn.4411 | NW_004082010 | 12672-13242 |
| TRDV1S19 | F | ChrUn.4604 | NW_004082203 | 3269-3825 |
| TRDV1S20 | F | ChrUn.4908 | NW_004082507 | 6129-6701 |
| TRDV1S21 | P | ChrUn.4908 | NW_004082507 | 10671-11205 |
| TRDV1S22 | F | ChrUn.4908 | NW_004082507 | 38638-39106 |
| TRDV1S23 | F | ChrUn.4908 | NW_004082507 | 50996-51569 |
| TRDV1S24 | F | ChrUn.5627 | NW_004083226 | 4865-5392 |
| TRDV1S25 | F | ChrUn.5967 | NW_004083566 | 6905-7461 |
| TRDV2 | F | Chr7 | NC_019464 | 22270939-22271510 |
| TRDV3 | F | Chr7 | NC_019464 | 22143095-22143620 |
| TRDV4 | F | Chr7 | NC_019464 | 21953950-21954534 |
| TRDD1 | F | Chr7 | NC_019464 | 22045175-22045186 |
| TRDD2 | F | Chr7 | NC_019464 | 22029306-22029320 |
| TRDD3 | F | Chr7 | NC_019464 | 22020455-22020465 |
| TRDD4 | F | Chr7 | NC_019464 | 22009347-22009360 |
| TRDD5 | F | Chr7 | NC_019464 | 21995102-21995114 |
| TRDD6 | F | Chr7 | NC_019464 | 21988913-21988921 |
| TRDD7 | F | Chr7 | NC_019464 | 21974176-21974186 |
| TRDJ1-1 | F | Chr7 | NC_019464 | 21973144-21973196 |
| TRDJ1-2 | F | Chr7 | NC_019464 | 21967336-21967384 |
| TRDJ1-3 | P | Chr7 | NC_019464 | 21965881-21965934 |
| TRDJ1-4 | F | Chr7 | NC_019464 | 21963501-21963559 |
| TRDC | F | Chr7 | NC_019464 | 21957629-21957891 |
| TRAJ1 | F | Chr7 | NC_019464 | 21877596-21877657 |
| TRAJ2 | F | Chr7 | NC_019464 | 21878564-21878629 |
| TRAJ3 | F | Chr7 | NC_019464 | 21879112-21879170 |
| TRAJ4 | F | Chr7 | NC_019464 | 21880078-21880138 |
| TRAJ5 | F | Chr7 | NC_019464 | 21883100-21883156 |
| TRAJ6 | ORF | Chr7 | NC_019464 | 21884274-21884335 |
| TRAJ7 | F | Chr7 | NC_019464 | 21884917-21884972 |
| TRAJ8 | F | Chr7 | NC_019464 | 21886463-21886522 |
| TRAJ9 | F | Chr7 | NC_019464 | 21887048-21887108 |
| TRAJ10 | F | Chr7 | NC_019464 | 21889305-21889364 |
| TRAJ11 | F | Chr7 | NC_019464 | 21889364-21889426 |
| TRAJ12 | F | Chr7 | NC_019464 | 21890368-21890425 |
| TRAJ13 | F | Chr7 | NC_019464 | 21890931-21890990 |
| TRAJ14 | F | Chr7 | NC_019464 | 21891781-21891830 |
| TRAJ15 | ORF | Chr7 | NC_019464 | 21892481-21892532 |
| TRAJ16 | F | Chr7 | NC_019464 | 21893364-21893423 |
| TRAJ17 | F | Chr7 | NC_019464 | 21894451-21894511 |
| TRAJ18 | P | Chr7 | NC_019464 | 21895868-21895920 |
| TRAJ19 | F | Chr7 | NC_019464 | 21896969-21897034 |
| TRAJ20 | F | Chr7 | NC_019464 | 21898318-21898375 |
| TRAJ21 | F | Chr7 | NC_019464 | 21899014-21899068 |
| TRAJ22 | F | Chr7 | NC_019464 | 21900541-21900603 |
| TRAJ23 | F | Chr7 | NC_019464 | 21901657-21901719 |
| TRAJ24 | F | Chr7 | NC_019464 | 21902102-21902161 |
| TRAJ25 | F | Chr7 | NC_019464 | 21902952-21903011 |
| TRAJ26 | F | Chr7 | NC_019464 | 21903249-21903308 |
| TRAJ27 | F | Chr7 | NC_019464 | 21905454-21905572 |
| TRAJ28 | F | Chr7 | NC_019464 | 21906104-21906176 |
| TRAJ29 | F | Chr7 | NC_019464 | 21907131-21907190 |
| TRAJ30 | F | Chr7 | NC_019464 | 21908143-21908199 |
| TRAJ31 | F | Chr7 | NC_019464 | 21910239-21910296 |
| TRAJ32 | F | Chr7 | NC_019464 | 21912040-21912109 |
| TRAJ33 | F | Chr7 | NC_019464 | 21912790-21912846 |
| TRAJ34 | F | Chr7 | NC_019464 | 21914217-21914273 |
| TRAJ35 | F | Chr7 | NC_019464 | 21915256-21915311 |
| TRAJ36 | F | Chr7 | NC_019464 | 21916743-21916803 |
| TRAJ37 | F | Chr7 | NC_019464 | 21917440-21917499 |
| TRAJ38 | F | Chr7 | NC_019464 | 21918926-21918987 |
| TRAJ39 | F | Chr7 | NC_019464 | 21919564-21919626 |
| TRAJ40 | F | Chr7 | NC_019464 | 21921521-21921577 |
| TRAJ41 | F | Chr7 | NC_019464 | 21923233-21923293 |
| TRAJ42 | F | Chr7 | NC_019464 | 21923698-21923763 |
| TRAJ43 | F | Chr7 | NC_019464 | 21924486-21924542 |
| TRAJ44 | F | Chr7 | NC_019464 | 21925784-21925845 |
| TRAJ45 | F | Chr7 | NC_019464 | 21926069-21926130 |
| TRAJ46 | F | Chr7 | NC_019464 | 21926915-21926975 |
| TRAJ47 | F | Chr7 | NC_019464 | 21927429-21927491 |
| TRAJ48 | F | Chr7 | NC_019464 | 21928173-21928229 |
| TRAJ49 | F | Chr7 | NC_019464 | 21930202-21930264 |
| TRAJ50 | F | Chr7 | NC_019464 | 21931090-21931145 |
| TRAJ51 | F | Chr7 | NC_019464 | 21931683-21931738 |
| TRAJ52 | P | Chr7 | NC_019464 | 21933059-21933117 |
| TRAJ53 | F | Chr7 | NC_019464 | 21934049-21934114 |
| TRAJ54 | F | Chr7 | NC_019464 | 21937256-21937321 |
| TRAJ55 | F | Chr7 | NC_019464 | 21937925-21937984 |
| TRAJ56 | F | Chr7 | NC_019464 | 21940674-21940735 |
| TRAJ57 | F | Chr7 | NC_019464 | 21941315-21941377 |
| TRAJ58 | F | Chr7 | NC_019464 | 21942539-21942601 |
| TRAJ59 | F | Chr7 | NC_019464 | 21943689-21943741 |
| TRAJ60 | F | Chr7 | NC_019464 | 21943934-21943990 |
| TRAJ61 | P | Chr7 | NC_019464 | 21944901-21944961 |
| TRAC | F | Chr7 | NC_019464 | 21874280-21874552 |

^a^ L-PART1/ V-exon for TRAV and TRDV genes and coding sequence for TRDD, TRAJ and TRDJ

^b^ nd: not defined (indicates that the nt sequence of the gene is incomplete and its functionality cannot be defined)
