# Supplementary material for: Sheep (Ovis aries) T cell receptor alpha (TRA) and delta (TRD) genes and genomic organization of the TRA/TRD locus
Source: BMC Genomics. 2015 Sep 18;16:709. doi: 10.1186/s12864-015-1790-z (PMC4574546; doi:10.1186/s12864-015-1790-z)
Supplement: Additional file 3: — Description of the TRAV/TRDV ORF and pseudogenes. (DOC 87 kb) [file 12864_2015_1790_MOESM3_ESM.doc]

**Additional file 3.** Description of the TRAV/TRDV ORF and pseudogenes

| **TRV**  **gene** | **Functionality** | **No initiation codon** | **No**  **L-exon** | **CYS 104**  **missing** | **Frameshift** | **Ins/Del in V-Ex** | **Stop codon** | **Defective splice sites** | **RS defect** |
| --- | --- | --- | --- | --- | --- | --- | --- | --- | --- |
| **TRA** |  |  |  |  |  |  |  |  |  |
| TRAV6 | P |  |  |  | ● |  |  |  | ● |
| TRAV8 | P |  |  |  |  |  | ● |  |  |
| TRAV9S1 | P |  |  |  |  |  | ● |  |  |
| TRAV14S1 | ORF |  |  |  |  |  |  |  | ● |
| TRAV22S1 | P |  |  |  |  |  |  | ● |  |
| TRAV22S4 | P |  |  |  | ● |  |  |  |  |
| TRAV22S5 | ORF |  |  |  |  |  |  |  | ● |
| TRAV23S1 | P |  |  |  | ● |  |  |  | ● |
| TRAV23S2 | P |  |  |  |  |  |  | ● |  |
| TRAV25S1 | P |  |  |  |  |  | ● |  |  |
| TRAV34 | P | ● |  |  | ● |  |  |  |  |
| TRAV35 | P |  |  |  |  |  | ● |  |  |
| TRAV37 | P | ● |  |  |  |  |  |  | ● |
| TRAV39 | ORF |  |  |  |  |  |  |  | ● |
| TRAV42S1 | ORF |  |  |  |  |  |  |  | ● |
| TRAV42S2 | P |  |  |  | ● |  |  |  |  |
| TRAV42S3 | P |  |  |  |  |  |  | ● |  |
| TRAV43S2 | P |  |  |  | ● |  |  |  |  |
| TRAV43S5 | P |  |  |  |  |  |  | ● | ● |
| TRAV44S1 | ORF |  |  |  |  |  |  |  | ● |
| TRAV44S4 | P |  |  |  | ● |  |  |  |  |
| TRAV44S7 | P |  |  |  | ● |  |  |  | ● |
| TRAV45S5 | P |  | ● |  |  |  |  |  |  |
|  |  |  |  |  |  |  |  |  |  |
| **TRD** |  |  |  |  |  |  |  |  |  |
| TRDV1S4 | P |  |  |  |  |  | ● |  |  |
| TRDV1S7 | P |  |  |  |  | ● |  |  | ● |
| TRDV1S8 | P |  |  |  |  |  |  | ● |  |
| TRDV1S9 | P |  |  |  |  | ● |  |  | ● |
| TRDV1S10 | P |  |  |  |  | ● |  |  |  |
| TRDV1S15 | P |  |  |  |  |  | ● |  |  |
| TRDV1S16 | P |  |  |  |  | ● |  |  |  |
| TRDV1S21 | P |  |  | ● |  |  |  |  |  |
|  |  |  |  |  |  |  |  |  |  |
