# Supplementary figures and images for: Sheep (Ovis aries) T cell receptor alpha (TRA) and delta (TRD) genes and genomic organization of the TRA/TRD locus
Source: BMC Genomics. 2015 Sep 18;16:709. doi: 10.1186/s12864-015-1790-z (PMC4574546; doi:10.1186/s12864-015-1790-z)

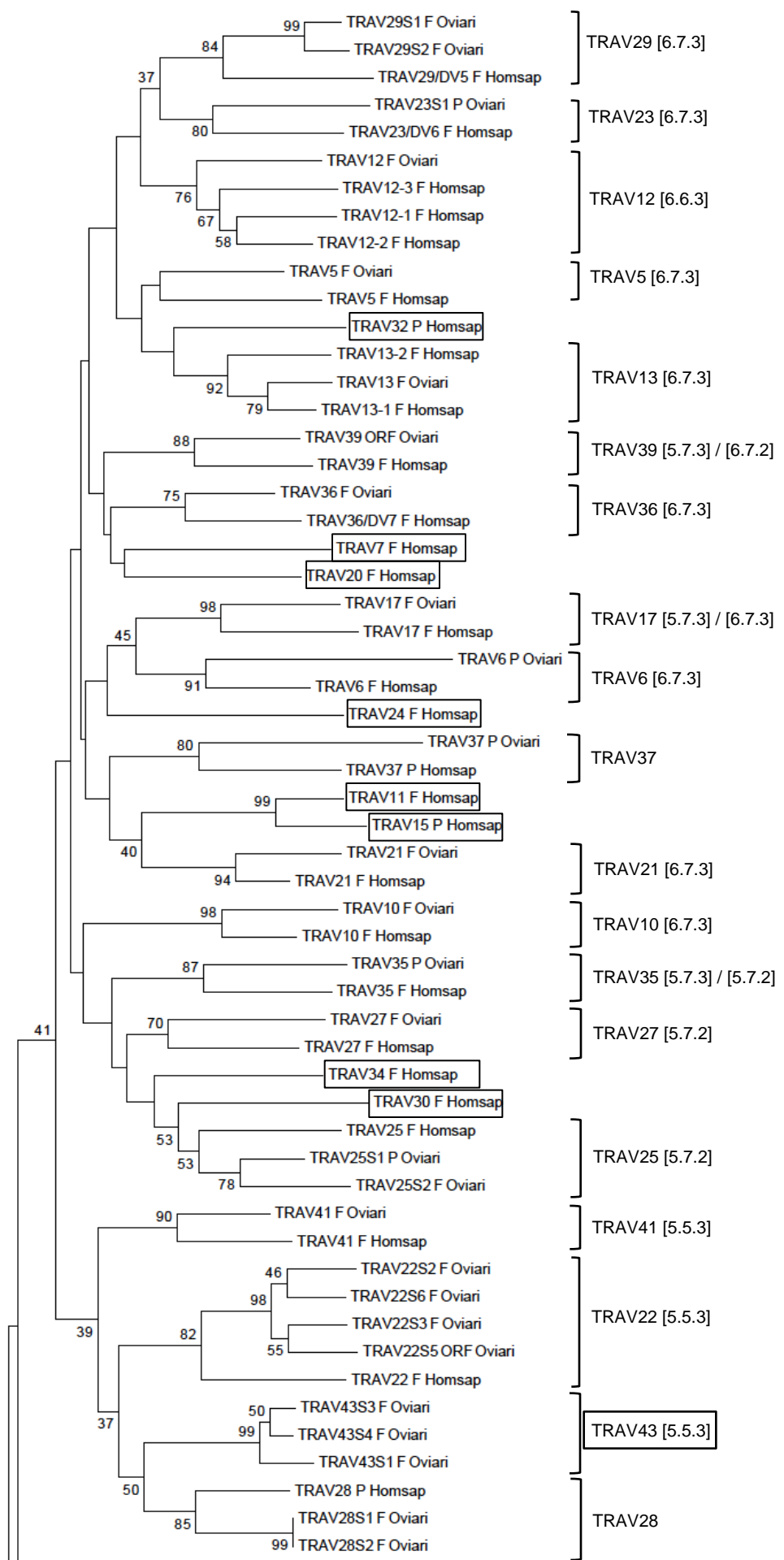

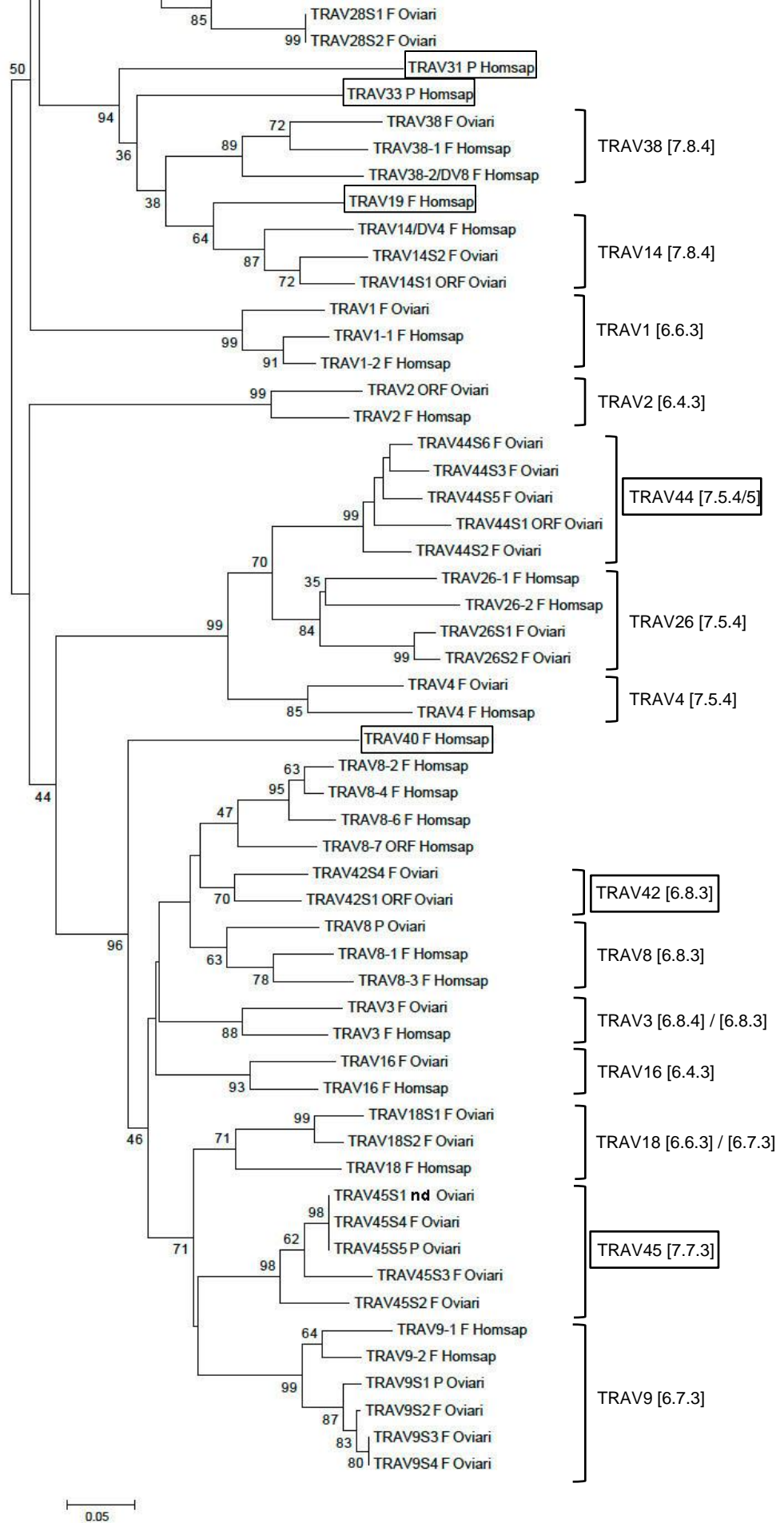

Supplement: Additional file 4: — The NJ tree inferred from the sheep and human TRAV gene sequences. The evolutionary analysis was conducted in MEGA5 [36]. The percentage of replicate trees in which the associated taxa clustered together in the bootstrap test (1000 replicates) is shown next to the branches [40]. The trees are drawn to scale, with branch lengths in the same units as those of the evolutionary distances used to infer the phylogenetic trees. The evolutionary distances were computed using the p-distance method [37] and are in the units of the number of base differences per site. The sheep TRAV subgroup classification is according to the corresponding human TRAV subgroups. Sheep-specific subgroups are numbered from TRAV42 (boxed in the right column). The TRAV genes found in humans and not in sheep are boxed in the tree. The gene functionality according to IMGT rules (F: functional, ORF: open reading frame, P: pseudogene) is indicated. For each subgroup, the CDR1-IMGT, CDR2-IMGT and CDR3-IMGT lengths are reported between brackets in the right column: when different between species, the first is for the human subgroup and the second is for the sheep subgroup. For TRAV42 to TRAV45, the CDR-IMGT lengths are for the sheep subgroup only (no corresponding subgroup in humans). The IMGT 6-letter standardised abbreviation for taxon is used: three letters for genus and three letters for species (Homsap, Oviari). (PPT 7.56 kb) [file 12864_2015_1790_MOESM4_ESM.pdf]

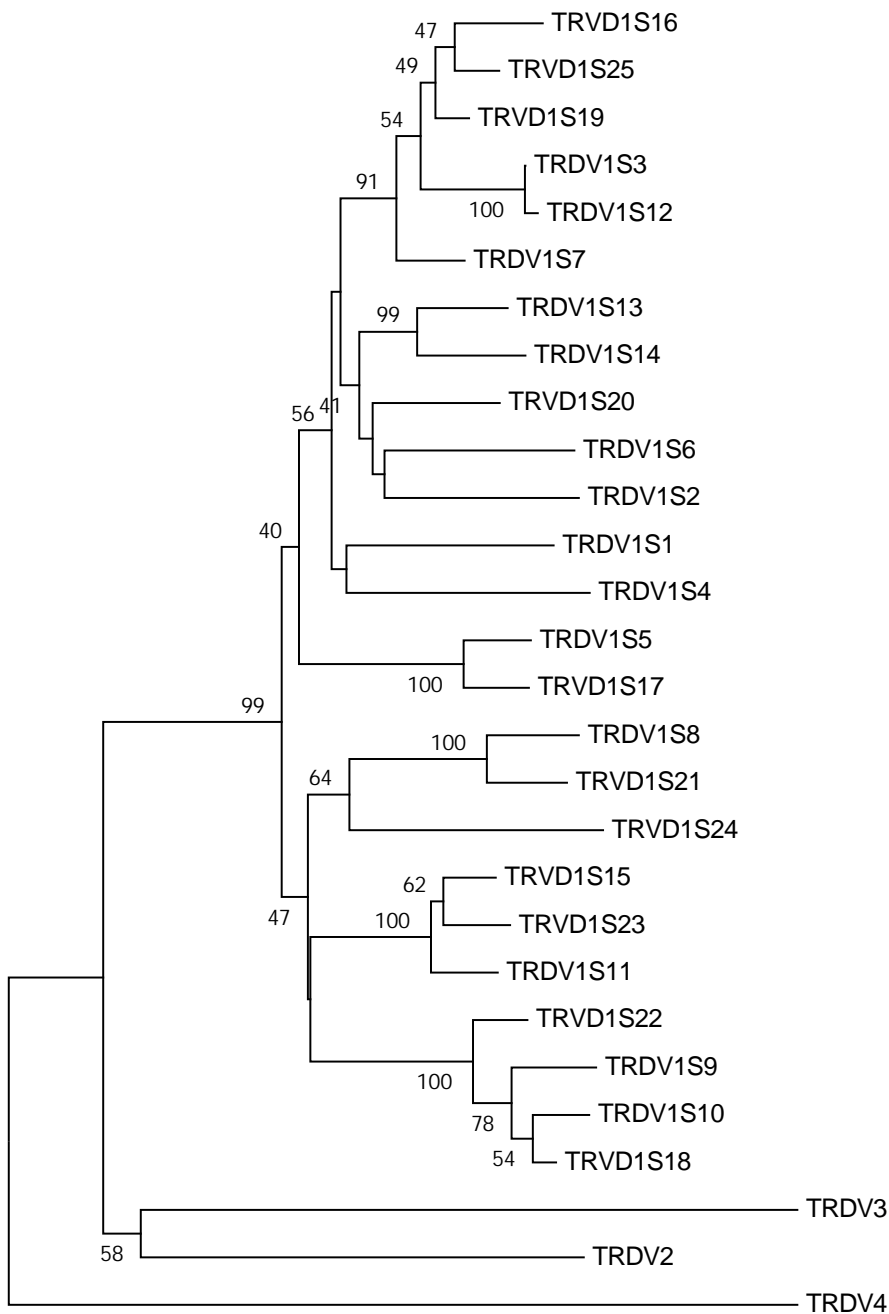

0.05

Supplement: Additional file 5: — Evolutionary relationship of the sheep TRDV genes. The TRDV nomenclature is according to IMGT-ONTOLOGY concepts of classification [28]. (PDF 7.56 kb) [file 12864_2015_1790_MOESM5_ESM.pdf]
