# Supplementary material for: Sheep (Ovis aries) T cell receptor alpha (TRA) and delta (TRD) genes and genomic organization of the TRA/TRD locus
Source: BMC Genomics. 2015 Sep 18;16:709. doi: 10.1186/s12864-015-1790-z (PMC4574546; doi:10.1186/s12864-015-1790-z)
Supplement: Additional file 6: — TRA genes. In a, the IMGT Protein display of the sheep TRAV genes. Only functional genes ORF and in-frame pseudogenes are shown. The description of the strands and loops and of the FR-IMGT and CDR-IMGT is according to the IMGT unique numbering for V-REGION [27]. The CDR-IMGT AA lengths are indicated in square brackets. nd: not defined (indicates that the AA sequence of the TRAV45S1 gene is incomplete and its functionality cannot be defined). In b, nucleotide and deduced AA sequences of the sheep TRAJ genes. The numbering adopted for the gene classification is reported on the left of each gene. The functionality is also reported. The consensus sequences of the J-heptamer and J-nonamer [41] are provided at the top of the figure and underlined. The donor splice site for each TRAJ is also shown. The canonical F/W-G-X-G amino acid motifs are underlined. In c, IMGT Protein display of the TRAC gene. Description of the strands and loops is according to the IMGT unique numbering for C-DOMAIN [42]. (ZIP 325 kb) [file 12864_2015_1790_MOESM6_ESM.pdf]

(a)

Gene	Functionality	L-PART1+L-PART2	FR1-IMGT (1-26) A (1-15)	FR1-IMGT (1-26) B (16-26)	CDR1-IMGT (27-38) BC (27-38)	FR2-IMGT (39-55) C (39-46)	FR2-IMGT (39-55) C' (47-55)	CDR2-IMGT (56-65) C'C" (56-65)	FR3-IMGT (66-104) C" (66-74)	FR3-IMGT (66-104) D (75-84)	FR3-IMGT (66-104) E (85-96)	FR3-IMGT (66-104) F (97-104)	CDR3-IMGT (105-117) FG (105-117)	
			1 10 15	16 **23** 26	27 38	39**41** 46	47 55	56 65	66 74	75 80 84	85 **89** 96	97 **104**	105	
TRAV1	F	MWNFFLLCVSLTVRGSA	GKGVEQ.PTELTAME	GASAQVN**C**TYQ	TSG......FNG	LF**W**YQQHD	GGAPVFLSY	NVL....DGL	ETR.....G	HFSSFLRRSD	AHSY**L**LLKELHM	KDFASYL**C**	AVI...	[6.6.3]
TRAV2	ORF	MALQSTLEAVWLGLLLIAES	KEQVFQ.PSTVVSLE	GAVAEIS**C**NHS	ISN......DYG	FF**W**YFHFP	GFAPRLLIE	GL......RP	SQQ.....G	RYNMTYER..	FSSS**L**LILQVQT	ADAGVYY**C**	AVR...	[6.4.3]
TRAV3	F	MASAHIWTLGILFTLSGLR	AQSVTQPEDEVPVAE	GDPVTVK**C**TYS	VSG......SPY	LS**W**YVQHR	NQGLQFLLK	YITG..DPLV	KGN.....Y	GFEAEFNESQ	TSFH**L**KKPSVLG	SDSAVYF**C**	AVS...	[6.8.3]
TRAV4	F	MRQMTRVTVLLTLGTLS	LAKTSQ.PIIIDSYE	GQEVNIP**C**NHT	TIAI.....SEY	IF**W**YRQFP	NQGPQFVIQ	GYT.....TT	VEN.....E	VASLLIPPDR	KFST**L**SLPRASL	GDAAVYY**C**	IVRD..	[7.5.4]
TRAV5	F	MKTPMGLWFLFLWLQLDYKSR	GEKVEQYPSFQSVQE	GDNCVIN**C**TYT	DSA......SAY	FV**W**YKQEP	GKGLQLLIH	TLSN...VDK	KEG.....Q	GLIVLLNKKN	KHLS**L**NITATHP	GDSATYF**C**	AAR...	[6.7.3]
TRAV8	P	MLLLLISILGMIFAPRDS*	GQLVTQPDDPVLVSE	GTSLELK**C**NYS	YGA......TPY	LF**W**YVQYP	GQRPQLLLK	YFSG..NTMV	QGI.....R	GFVAEFRSSD	FSFN**L**RKFSAHW	SDSAEYF**C**	ALS...	[6.8.3]
TRAV9S1	P	MNYPLGSVIVLFLMFGGSN	GDSVTQPKG*VTLLE	GESSTVN**C**SYE	TEQ......YPA	LF**W**YVQYL	GEGPQLLLR	AQRD...NKK	GSN.....K	GFEATYNTET	TSFH**L**EKALVQE	SDSAVYY**C**	ALS...	[6.7.3]
TRAV9S2	F	MSSSPGLVTVILLMLRQTH	GNSVTQMDGQVSRSE	GTSVTIN**C**TYS	ASG......YPA	LF**W**YVQYP	GEGPQLLLK	ATKA...GDK	GTN.....K	GFEATYNTET	TSFH**L**EKASVQE	SDSAVYY**C**	ALS...	[6.7.3]
TRAV9S3	F	MSSSPGLVTVILFLLGQTR	GDSVSQMDGQVTLLE	GATLTVN**C**TYS	AIG......YPT	LF**W**YVQYP	GEGPQLLLK	ATKA...NDK	GTN.....E	GFEATYDAKT	TSFH**L**EKASVQE	SDSAVYH**C**	ALS...	[6.7.3]
TRAV9S4	F	MSSSPGLVTVILFLLGQTR	GDSVSQMDGQVTLLE	GATLTVN**C**TYS	AIG......YPT	LF**W**YVQYP	GEGPQLLLK	ATKA...NDK	GTN.....E	GFEATYDAKT	TSFH**L**EKASVQE	SDSAVYH**C**	ALS...	[6.7.3]
TRAV10	F	MEMHVRSSLVVLWLHFCCVSG	KNQVEQSPPSLAVLE	GENCTFQ**C**NYT	VSP......FNT	LR**W**YTQDT	GRGLVSLIT	MTYS...DNK	KSN.....G	RYTATMDATA	KHSF**L**HLTAAQL	SDLAFYI**C**	VVG...	[6.7.3]
TRAV12	F	MKSSRVLLVILWVHLISVSSQ	QNTVEQSPASLPVPE	GVIASLG**C**TYS	DSY......SQY	FT**W**YRQYP	GKGPEFLLQ	VYA....DKD	KEE.....G	KFTVQSNKTN	KRVS**L**RIRDSEP	SDSATYL**C**	AVS...	[6.6.3]
TRAV13	F	MKTPIGALITFLWLQLDCVSL	GNKVEQSPSTLNVQE	GNSSVIT**C**TYT	NGY......SEY	FP**W**YKQEP	GKGPQLLIA	IRSN...KDK	EED.....Q	RLTVLLNKTA	KRLS**L**HIAATEA	GDSAVYF**C**	AAG...	[6.7.3]
TRAV14S1	ORF	MLLSSLLRVVVASLCLGSTV	AQKVTQDPQPILVQE	KEAVTLD**C**TYD	TSDS.....SYS	LF**W**YKQPN	SGAMIFLLS	QDSY..GKQN	ASE.....G	RYSLNFQKAS	KSIT**L**VISASQL	EDSAVYF**C**	ALRE..	[7.8.4]
TRAV14S2	F	MLLSSLLRVVVASLCLGSSI	AQKVTQDQPPMSVQE	KENVTLD**C**TYD	ISIT.....TYS	LF**W**YKQPS	SGVMTFLIR	QDSS..NKPN	ATE.....G	RYSLNFQKAG	KFIT**L**TISASQL	EDSAVYF**C**	ALSE..	[7.8.4]
TRAV16S1	F	MKRALISVLVMIFTLGGTR	AQTVTQPESHISVSE	GDPVQVK**C**SYS	YSG......SPV	LF**W**YVQYP	RQHLQLLLK	HT......SK	ESI.....Q	GFTAELSRAE	ASFH**L**KKPSTQE	EDSAVYY**C**	ALG...	[6.4.3]
TRAV17	F	MEKLLALSLVILWLQLAKVN	SQQGEQNLQTLSIQE	GENITMN**C**SYK	SIT......ITA	LQ**W**YRQDS	RRGFVHLIL	MRSN...ERQ	KHS.....G	RLHFTLDSSI	KSSS**M**SITASQA	EDTATYF**C**	APD...	[6.7.3]
TRAV18S1	F	MLSALCLGLVTLLIMRTS	ADSVTQTEGVVTLPE	MASLTLK**C**TYQ	SSY......SVS	LF**W**YVQYQ	NKELELLLK	SLLG...NQK	VTS.....R	GFEATHISSD	SSFH**L**QKSSVKT	SDSAVYY**C**	ALS...	[6.7.3]
TRAV18S2	F	MLSALCLGLVIFMIMRGTS	GDSVTQTEGVVILPE	KASLTLK**C**TYQ	SSY......SDF	LF**W**YVQYK	NKELELLLK	SSLD...NQK	VTS.....R	GFEATHISSD	SSFH**L**QKSSVQT	SDSAVYY**C**	ALS...	[6.7.3]
TRAV21	F	MDTSLSLLILWLQLDWSSS	KQDVSQSPEALSVRE	GDSLVLN**C**SYT	DSA......LYF	LQ**W**FRQDP	GKGLISLLS	IQAN...QKE	QTS.....G	RITVSLDKSS	RHSA**L**YIATSQH	SDSTTYL**C**	AVR...	[6.7.3]
TRAV22S2	F	MKRLVGTVLGLLLAWVCCVR	GVDVEQSPPALTPQE	GASSTLW**C**NFS	TLA.......DT	VR**W**YLQKP	GGRLIHLIY	IPS.....GT	RQE.....G	RLNATAVPKE	RRSS**L**HISSLRT	TDSGTYF**C**	AVR...	[5.5.3]
TRAV22S3	F	MKRLAGAVLGLLFAQVCGVR	GVDVEQSPPALSLQE	GASYTLQ**C**NFS	TSP.......QS	VN**W**YLQNS	EGHLIQLFY	IPS.....GT	KQD.....G	RLKATTVPAE	RRSS**L**HISSSQT	TDSGTYF**C**	AAQ...	[5.5.3]
TRAV22S5	ORF	MKRLAGAVLGLLFAQVCCVR	GVDVEQSPPALSLQE	GASYTLR**C**NFS	TFP.......QS	VN**W**YLQNP	GGHLIHLFY	IPS.....GT	KQD.....E	KLNTVTVLTE	RRSS**L**HVSSSQT	TDSGTYF**C**	AVQ...	[5.5.3]
TRAV22S6	F	MKRLAGTVLGLLFAQVCCVR	GLDVEQSPPALSLQE	GASHMLR**C**NFS	ASV.......SN	VQ**W**YLQNP	SGRLIHLFN	IPS.....GT	KQD.....G	RLNATTIPTE	RRSS**L**HVSSSQT	TDSGTYF**C**	AAQ...	[5.5.3]
TRAV25S1	P	MLLLAPVLILWIQIS*MN	GQQIKHFPEFLLLQE	GENFTTY**C**NSS	STF.......YN	LQ**W**YKQRP	GGSPVFLMI	LTKP...GEA	KTE.....Q	RLTGRLGETR	QHSS**L**HLAAAQL	SDAGTYF**C**	AE....	[5.7.2]
TRAV25S2	F	MLLIAPVLILWMQIPQMN	GQQIKHFPESLLLQE	GENFTTY**C**NFS	STF.......YN	LQ**W**YKQRP	GGSPVLLMI	LAKG...GEV	KTQ.....Q	RLTVRFGESR	QHSS**L**HLTATQP	SDVGTYF**C**	AT....	[5.7.2]
TRAV26S1	F	MRLVTGITLLLALGVLG	DSKTTQ.PNSVEGTE	EEPVHLP**C**NHS	TISG.....SEY	VY**W**YRQIP	RQGPEYLIH	GLN.....NN	VTN.....R	MASLSIAKDR	KSST**L**VLPQVTL	RDTAVYY**C**	ILRD..	[7.5.4]
TRAV26S2	F	MRLVTGITLLLALGVLG	DTKTTQ.PNSVEGAE	EEPVHLP**C**NHS	TISG.....PEY	IY**W**YRQIP	HQGPEYLVH	GLN.....NN	VTN.....R	MASLSIAKDR	KSST**L**VLPQVTL	RDTAVYY**C**	ILRE..	[7.5.4]
TRAV27	F	MLQKLLVLVFWIQLAWES	TQQLEQNPKFLRIQE	GGNVTLY**C**NSS	STF.......TF	FQ**W**YRQKP	EEGPVLLVT	LTKP...KEM	KEQ.....K	RIRAEFGEAR	KDSS**L**LITAAQP	GDAGTYL**C**	AG....	[5.7.2]
TRAV28S1	F	MKTQRRVLLSLLWIQICWLSV	QMKVEQSPGVLTLQE	GRNSSLI**C**NYS	ISI.......RS	VQ**W**FQQNP	DGRLISLFY	IAS.....GM	QQK.....G	RLKSTINSKE	RYSQ**L**YIRDSQP	GDSATYF**C**	AVE...	[5.5.3]
TRAV28S2	F	MKTQRRVLLGLLWILICCLRV	QMKVEQSPGVLTLQE	GRNSSLI**C**NYS	ISM.......TS	VQ**W**FQQNP	DGHLISLFY	IAS.....GM	QQK.....G	RLKSTINSKE	RYSQ**L**YIRDSQP	GDSATYF**C**	AVE...	[5.5.3]
TRAV29S1	F	MSKFSGTSLLILWLQSDWVKSQQNSA	QQQVKQNPPSLSVTE	GGISILN**C**DYD	NTI......LDY	FQ**W**YRKYP	AKSPTSLIS	ISSV...LEK	NED.....G	RFTVFHNRSA	KHLS**L**HISASQP	GDSALYL**C**	AAS...	[6.7.3]
TRAV29S2	F	MSKLLGTSLLILWLQSDWVKSQQKNGD	QQKVKQNPPSLSVTE	GGISILN**C**DYD	DIM......FNY	FQ**W**YRNYP	SKSPTFLIS	IGSV...LEK	NED.....G	RFTVFHNRSA	KHLS**L**HISASQP	GDSALYL**C**	AAS...	[6.7.3]
TRAV35	P	MFLED*LIILWMHLTCVS	AQQLNQSPQSMSIQE	GEDLSMN**C**NSS	STL.......NL	LL**W**YKQDA	GEGLILLIK	LLKG...GEL	ARN.....G	KLTAQFGGTR	KDSL**L**NNSAFEP	KDGGTYF**C**	AG....	[5.7.2]
TRAV36	F	METWPRALLGIFWLQLSLVKS	DDNVMQSPSSLIVLE	GSNATLS**C**SYK	VTN......FQS	LH**W**YKQEE	KVP.TFLFV	LIST...GIE	KTS.....G	RLKGTLDRKE	LLST**L**YITVTKP	GDSATYL**C**	AVE...	[6.7.3]
TRAV38	F	MTLVSLLWAVLVSMCLGPSV	AQTVTQPQPEESVQE	ADTVTLD**C**TYT	TSES.....DCY	LF**W**YKQPP	SGEMVFIIH	QYAY..EQQN	STN.....D	RYSVNFQKES	KAFS**L**RISDSQL	EDAAKYF**C**	AYSG..	[7.8.4]
TRAV39	ORF	MKKLLATILWLQLSWLN	ADLKVEQSPPFLIIR	ERQAGIN**C**DHS	VTTS......DT	LL**W**YRQDQ	GKSLESLFL	LMSN...GAV	RKK.....G	GLTASLDTKA	RRSP**L**HITASHP	GLSATYF**C**	AM....	[6.7.2]
TRAV41	F	MVKTQQVSLAILWLQLHWVSG	KNGVEQSPRYLSAQE	GDLITIN**C**NYL	EGM.......TT	LQ**W**LQQNP	GGSIISLLT	LSL.....EM	KKK.....G	RVSATINRRE	RYSS**L**NITAPQP	TDSAIYF**C**	AVE...	[5.5.3]
TRAV42S1	ORF	MSLVPITVLITLLALRGAG	AQSVTQPDSYITVSE	RSRLELR**C**NYS	SPV......SLY	LF**W**YMQYP	NQGLQLLLK	YISG..EGLV	SGI.....K	GFKAEFINET	T.FH**L**EKPLAHW	RDSAKYF**C**	ALD...	[6.8.3]
TRAV43S1	F	MKRKWGILLGLLCIQISWVR	GVKVEQSPSVLSLQE	GANSTLW**C**NFS	DTV.......SS	VQ**W**FQQNP	EGSIISLFF	IAS.....GT	KQN.....E	RLSSTLNSKE	RYST**L**HITASQL	EDAATYL**C**	AAE...	[5.5.3]
TRAV43S3	F	MKRKWGALLGFLWVQICWVR	GVKVEQRPSILSLQE	GANSTLW**C**NFS	DTV.......ST	VQ**W**FQQNP	GGSLTRLFV	IAS.....GT	KQN.....E	RLSSTVNSKE	QYST**L**HITASQL	EDAATYL**C**	AVE...	[5.5.3]
TRAV43S4	F	MKRRWGTLLGFLWIQTCWVR	GLKVEQSPSILSLQE	GANSTLW**C**NSS	DTV.......KS	VQ**W**FQQNP	RGSLITLFF	IAS.....GM	KQN.....E	RLSSTVNSKE	QYST**L**HITASQL	EDAATYL**C**	AVE...	[5.5.3]
TRAV44S1	ORF	MRLVIGVTVFLTLGTVI	DAKTTQ.PSSMDCAE	EEDVTLS**C**SHS	TIGG.....NDY	IH**W**CQQNP	NQSPQYVIH	GLR.....GT	VNS.....S	MASLHIASDK	NSST**L**VLPQVIL	RDTAVFY**C**	VLREA.	[7.5.5]
TRAV44S2	F	MRLVTGVTVLLTLGIMV	DVEITQ.LSSMDCAE	GEDVNLP**C**NHS	TIDA.....LDY	IH**W**YRQTP	NQSPQYVIH	GFR.....DT	VNS.....S	MGSLNIASDR	KSST**L**VLSQVTL	RDTAVYY**C**	VLRE..	[7.5.4]
TRAV44S3	F	MRLVTGVTVFLTLGTVI	DAKTIQ.PSSMDCAE	EEDVNLP**C**NHS	TISG.....NEH	IH**W**YRHNP	NQSPQYVIH	GLR.....GT	VNS.....S	MASLHIASDR	KSST**L**VLPQVTL	RDTAVYY**C**	ILRE..	[7.5.4]
TRAV44S5	F	MRLLIGVIVFLTLGNVI	DAKTTQ.PSSMDYAE	EEDVTLT**C**SHS	TIGG.....DVY	IH**W**CQQNP	SQSPQYVIH	GLR.....GT	GNS.....S	MASLTIDSDR	KSST**L**VLPQVTL	RDAAVYY**C**	IVRE..	[7.5.4]
TRAV44S6	F	MRLVTVVTVFLTLGKWTMI	DTKTTQ.PSSVDCAE	GENVNLP**C**NHS	TIGG.....DDY	IH**W**YRQNP	NQSPQYVIH	GFR.....DT	VNG.....S	MASLTIASDR	KSST**L**VLPQVTL	RDAAVYY**C**	IVRE..	[7.5.4]
TRAV45S1	nd				Y	LF**W**YVQHL	SKAPQLLLK	GLTA...DKK	VEH.....E	GFQATLVQSD	RSFH**L**QKRAVQA	SDSAVYY**C**	ALS...	[?.7.3]
TRAV45S2	F	MHSATHSVLLIILIFRGTN	GDSVNQTEGSVTVSE	GALMTLN**C**TYQ	TAG.....LTPY	LY**W**YVQHL	SKAPRLLLK	GSER...DPK	PKS.....E	GFQATLVQSD	RSFH**L**QKLAVQT	SDSAVYY**C**	ALS...	[7.7.3]
TRAV45S3	F	MHPVTHSVLLIILVLGGTN	GDSVNQTEGPVTVSE	GALMTLN**C**TYQ	ATY.....SIVY	LF**W**YVQHL	NKAPRLLLK	GSMS...DLK	PKS.....E	GFQATLVQSD	RSFH**L**QKRAVQA	SDSAVYY**C**	ALS...	[7.7.3]
TRAV45S4	F	MHPVTHSVLIILIFRGTN	GDSVDQTEGPVTVSE	GALMTLN**C**TYQ	ATY.....SNVY	LF**W**YVQHL	SKAPQLLLK	GLTA...DKK	VEH.....E	GFQATLVQSD	RSFH**L**QKRAVQA	SDSAVYY**C**	ALS...	[7.7.3]
TRAV45S5	P	---------------GGTN	GDSVNQTEGPVTVSE	GALMTLN**C**TYQ	ATY.....STVY	LF**W**YVQHL	SKAPQLLLK	GLTA...DKK	VEH.....E	GFQATLVKSD	RSFH**L**QKRAVQA	SDSAVYY**C**	ALS...	[7.7.3]

(b)

Gene name	Functionality	J-NONAMER GGTTTTTGT	12-spacer	J-HEPTAMER CACTGTG	TRAJ	5'splice donor
TRAJ1	F	gctttctgt	aatgaagacagt	gagagtg	AGATGGAGGTGTTGCCTCCCAGATGCAGTTTGGCAAGGGAACCAGAGTCTCCATCACTCCAG	gtatgt
					D G G V A S Q M Q F G K G T R V S I T P	
TRAJ2	F	agtttctgt	aatggtatcccc	agcagtg	TGGATACAGGAGGAGTGATGAGTAAACTCACGTTTGGGAAAGGAACCCAAGTGTCCATAATATCTG	gtgagt
					D T G G V M S K L T F G K G T Q V S I I S	
TRAJ3	F	ggttattgc	aaagaccttacc	ctcggtg	GAGATTCAGCATTGATAAGTTAAACTTTGGAGCAGGGACCAGACTGAGTGTCCAACCAA	gtaagt
					R F S I D K L N F G A G T R L S V Q P	
TRAJ4	F	ttcttgtaa	agcatcctccta	gtgggtg	TCATCTGGTGGCTACAATAAGCTGACCTTTGGACCCGGGACCAGGCTGACTGTACACGCAC	gtgagt
					S S G G Y N K L T F G P G T R L T V H A	
TRAJ5	F	tgtttgtac	tgcactgtggca	gggtgtg	GGCCCAGGCAGCAGAGCACTCACTTTGGGGGTGGAGCAAGACTCCGCAGACATCCAA	gtgagt
					P R Q Q S T H F G G G A R L R R H P	
TRAJ6	ORF(1)	ggttttagc	aagggctttcct	tgctgtg	TGTATCAAGACGAAACTATGGACTTATACTTGGAAGAGGGACCAAGCTTGTTGTTCACCCAC	gtgagt
					V S R R N Y G L I L G R G T K L V V H P	
TRAJ7	F	tttttgtaa	tgcacttaccca	gagtgtg	TGATTATGGGAACAGAGTCACTTTTGGAAACGGGACTCAAGTGCTGGTCACACCAA	gtaagt
					D Y G N R V T F G N G T Q V L V T P	
TRAJ8	F	ccattttgt	atagagttatgt	cagagtg	TGAACACAGGTTATCAGAAATTCATATTTGGAACTGGCACCCAACTTGTGATCAACCCAA	gtaagt
					N T G Y Q K F I F G T G T Q L V I N P	
TRAJ9	F	ccattttgt	cacagcacaaat	cactgtg	GGAAATACGGGAGGCTTCAAAGTTGTCTTTGGGACAGGAACAAAGCTATTTGTTGAAACAA	gtaagt
					G N T G G F K V V F G T G T K L F V E T	
TRAJ10	F	ccattttgt	atagagttatgt	cagagtg	TGAACACAGGTTATCAGAAACTCGTATTTGGAACTGGCACCCAACTTTTAATCACCCCAA	gtaagt
					N T G Y Q K L V F G T G T Q L L I T P	
TRAJ11	F	agtttattg	tgaggcatcaga	cactgtg	GGAATTGGGGAGGAGGAAGCAAACTCACCTTTGGGAAAGGCACCCACCTAAAAGTGAAGCTGG	gtaagt
					N W G G G S K L T F G K G T H L K V K L	
TRAJ12	F	ttttggtat	gggggtttgcta	cagtgtg	AATTCAGGATACAACACACTTACTTTTGGAAAGGGCACGGTGCTTCTTGTCTTTCCAG	gtaaat
					N S G Y N T L T F G K G T V L L V F P	
TRAJ13	F	tatttttga	ctgactaagaaa	cactgtg	GGAAGGGAGGAGGCTATACGTGGACCTTCGGAAGCGGGACTAGACTGCTGGTCAGGCCTG	gtgagt
					K G G G Y T W T F G S G T R L L V R P	
TRAJ14	F	attctgtat	aggcctgcattg	cagcgtg	AATCCTAAAGGTTGCCTTTGGAACTGGAAGGATACTTCAAGTCACCCTAA	gtaaat
					I L K V A F G T G R I L Q V T L	
TRAJ15	ORF(2)	aattattgt	caggcagcacgg	tgctgtg	ATTTATAACACATTCATCTTTGCAAGTGGGACAAGATTCTCAGTAAAACCCA	gtaagt
					I Y N T F I F A S G T R F S V K P	
TRAJ16	F	ggtatttgc	agcaccttgttt	cactgtg	CCTACCGGGCAGGAAATGCACTGGTCTTTGGGAAAGGAACCACCGTATCAGTGCATCCCA	gtaagt
					Y R A G N A L V F G K G T T V S V H P	
TRAJ17	F	ggtttttgt	ggtgaaatagat	cactgtg	GGGTTTTCAAGTGGCCAGAAGTTGGTTTTTGGAAGTGGGACCATGCTAAAGGTGAATCTTA	gtaagt
					G F S S G Q K L V F G S G T M L K V N L	
TRAJ18	P(3)	ggtttttgc	tgggcctcaaat	cactgtg	TGACCACCACTGCAGGGAGCAAGCTAACCTTTGGAGAAGGAACCAGGGTGACA	gtcaag
					T T T A G S K L T F G E G T R V T	
TRAJ19	F	ggttcatgt	aaagggggccgg	cactgtg	TCGATAGAGGCTCAAGTCTGGGGAAGTTCTACTTTGGAAGAGGAACCCGGCTAACTGTACAGCCTG	gtgagt
					D R G S S L G K F Y F G R G T R L T V Q P	
TRAJ20	F	ggtttgcgt	aggaagacgtag	cactgtg	ACTCTAAATAACTACAAGTTCACCTTTGGATCAGGAACCACAGTAACTGTGAGAGCAA	gtaagt
					T L N N Y K F T F G S G T T V T V R A	
TRAJ21	F	actttttgt	aatggtagtaaa	catggtg	ACAACACCAACAGATTTTACTTCGGATCTGGGACAAAACTCAGTGTAAAGCCAA	gtaagt
					N T N R F Y F G S G T K L S V K P	
TRAJ22	F	ggtttttgt	tgttgggcttat	catagtg	CTTCCTCTAGTTCAAGCTGGCACCTGACCTTTGGATCTGGGACCCAACTGACTGTTGTACCTG	gtaggc

					S S S S S W H L T F G S G T Q L T V V P	
TRAJ23	F	tgtttttga	caggctgtataa	cactgtg	TGAATTATAACCAGGGAGGAAAGCTTATCTTCGGACAGGGAACCGAGTTATCTGTGAAGCCCA<br>N Y N Q G G K L I F G Q G T E L S V K P	gtaagt
TRAJ24	F	attttgtag	aggtgttcgtca	cagtgtg	ACAACTGACGGCTGGGGGAAATTGAATTTTGGAGCGGGGACCCAGGTTGTGGTTACCCCAG<br>T T D G W G K L N F G A G T Q V V V T P	gtaagc
TRAJ25	F	ggtttttga	tgctgagataat	cactgtg	GGGAAGGACAAGGCTTCTCCCTCATCTTTGGGAAGGGGACAAGGCTGCTTGTCAAGCCGA<br>E G Q G F S L I F G K G T R L L V K P	gtaagt
TRAJ26	F	gatttttgt	aaagcccagggg	cactgtg	GGAATAACTATGGCCAGGGTTTGATCTTCGGTGGAGGAACCAGATTGTCTGTGCAGCCCC<br>N N Y G Q G L I F G G G T R L S V Q P	gtaagt
TRAJ27	F	ggttattgc	aatagcactgag	cactgtg	TAACACCGGCACAGGCAAATTAACCTTTGGGGACGGGACCGCGCTCACTGTGAAGCCAA<br>N T G T G K L T F G D G T A L T V K P	gtaagt
TRAJ28	F	ggtttttgc	aaagaaaggaaa	ctctgtg	CCTACTCTGGGGCTGGGAGTTACCAACTCAACTTCGGGAAGGGCACCAAACTCTTGGTCACACCAA<br>Y S G A G S Y Q L N F G K G T K L L V T P	gtaagt
TRAJ29	F	agtttttgt	gatgaaggcaat	cactgtg	GGGATTCAGGAGACAGGCGACTTGTCTTTGGAAAAGGCACAAGACTTGCTGTGACTCCAA<br>D S G D R R L V F G K G T R L A V T P	gtaagt
TRAJ30	F	tttttgttg	tggccccaatca	cagtgtg	AACAAACCACTGGCAAAATTGTCTTTGGAAGAGGGACTCAGCTTCATGTTCTCCCCA<br>Q T T G K I V F G R G T Q L H V L P	gtaagt
TRAJ31	F	ggtttcagt	aaaagcaggaaa	tgctgtg	GGGACTGACAATACCAGAATCTTCTTTGGAACTGGAACCCAGGTGGTGGTAAAGCCCA<br>G T D N T R I F F G T G T Q V V V K P	gtaagt
TRAJ32	F	ggttgttgt	aaggcactgaaa	ggctgtg	TGAATTATGGAGGCGCTGCCAACCAGCTCACCTTTGGGACTGGCACGTTGCTTTCTGTCAAGCCAAGTAC<br>N Y G G A A N Q L T F G T G T L L S V K P S	gtgagt
TRAJ33	F	ggtttttgt	taaggtttttgt	ctctgtg	TGGATGGCAACTATCGGTTGATCTGGGGCTCTGGGACCAAGCTAATTATAAAGCCAG<br>D G N Y R L I W G S G T K L I I K P	gtaagt
TRAJ34	F	ggtttttgt	aggccttagtat	cactgtg	TCTCCAACAGCAACAAACTCATCTTTGGGGCTGGAACCAGATTACAAGTTTTTCCAA<br>S N S N K L I F G A G T R L Q V F P	gtaagt
TRAJ35	F	ggtttttgt	agtggagtcagc	cattgtg	GACCTTTGGGAATGTGCTGCATTGGGGGTCTGGCACTCAAGTGATTGTTATACCTC<br>T F G N V L H W G S G T Q V I V I P	gtaagt
TRAJ36	F	agtttttgt	actgggcagaaa	cactgtg	ATAACTGCTGGAGCAAATCGGCTTGTCTTTGGGACAGGAACAAGCCTCACCGTCCTCCCCC<br>I T A G A N R L V F G T G T S L T V L P	gtaagt
TRAJ37	F	ttcttgtaa	agtagagcatta	cagtgtg	GCTCAGGAAACACAGGAAGACTCACCTTTGGGCAGGGGACAGTGTTACAAGTAAAACCAG<br>S G N T G R L T F G Q G T V L Q V K P	gtaggt
TRAJ38	F	ggtttctgt	aaagctttccat	gactgtg	TAATATTGGCAACAACCGTAAGCTGATTTGGGGACTGGGGACAAGTCTGGCAGTAAATCCAA<br>N I G N N R K L I W G L G T S L A V N P	gtgagt
TRAJ39	F	ggtttttgc	tgagctgaagat	cactgtg	TGAATAATAATGCAGGCAACGTGTTCACATTTGGAGGGGGAACAAGGTTAATGGTCAAACCTC<br>N N N A G N V F T F G G G T R L M V K P	gtgagt
TRAJ40	F	ggtttgtgt	agagccacgtag	cactgtg	ACTCAGGAAACTACAAATACGTCTTTGGAGCAGGCACTAAGCTGCAGGTTTTAACAA<br>S G N Y K Y V F G A G T K L Q V L T	gtgagt
TRAJ41	F	ggattttgt	ttagggaaggtg	cactgtg	GAACTCAATGCCGGCTACGTGCTCCGCTTTGGCCAAGGCACCTCAGTGCTGGTCACACCCG<br>E L N A G Y V L R F G Q G T S V L V T P	gtgagt
TRAJ42	F	gataattgt	aaagtcccatat	gactgtg	TGAATGTTGGCAGCAGCCAAGGAAAGCTCATCTTTGGAAAAGGCACTATGGTCTCTGTTAAACCAA<br>N V G S S Q G K L I F G K G T M V S V K P	gtaagt
TRAJ43	F	ggtttttgt	tagagggtgtac	tgctgtg	GCAGTTATAACAACGACCTGCGCTTTGGAGCAGGGACCAGACTGATGGTAAAACCAA<br>S Y N N D L R F G A G T R L M V K P	gtaagt
TRAJ44	F	ggtttctgt	catggagcatct	cacagtg	TAACACGGGCAGTGGCGGGAAAGTCATATTTGGGACTGGAACGAGACTTCAGGTCACCCTTG<br>N T G S G G K V I F G T G T R L Q V T L	gtaggt
TRAJ45	F	ggtttctgt	catggagcatct	cacagtg	TAACACGGGCAGTGGCGGGAAAGTCATATTTGGGACTGGAACGAGACTTCAGGTCACCCTTG<br>N T G S G G K V I F G T G T R L Q V T L	gtaggt

TRAJ46	F	tttatgtaa	agggttgacatg	gggtgtg	AATACAGGAGGAGGTAACAGACTCATCTTTGGAAAAGGGACTCAGCTCATCATCCAGCCTA	gtaagt
					N T G G G N R L I F G K G T Q L I I Q P	
TRAJ47	F	tgtttctgt	aaagctgctgac	aacagtg	AGAAGAGTGGCAGCGGAGACAGGCTGACTTTTGGGACCGGGACGCGTTTAGCAGTGAGGCCCA	gtaagt
					K S G S G D R L T F G T G T R L A V R P	
TRAJ48	F	tgtttttgt	agaggagtttga	tgctgtg	TGAATTATGGAAACACACTGATCTTTGGCTCAGGAACCACTTTGACAGTCAAGCCTC	gtgagt
					N Y G N T L I F G S G T T L T V K P	
TRAJ49	F	ggtttttgt	aatgatttaaaa	cactgtg	TGTCTAACTACCAAGCCAGTCAATTAAACTTTGGAACAGGAACTAGACTCACCATCACAGCCA	gtaagt
					S N Y Q A S Q L N F G T G T R L T I T A	
TRAJ50	F	ggtttttgt	tgagcttcctat	cacagtg	GAACAGCTACGGCCAGAACTATTTTGGGAGAGGGACAAGTTTGACAGTCATTCCAA	gtaagt
					N S Y G Q N Y F G R G T S L T V I P	
TRAJ51	F	agttattgc	aaagtgttcgat	ggctgtg	TGACAACTCCTACAAGTTGATGTTCGGGCAAGGGACGAGCTTATCAGTCATTCCAA	gtaagt
					D N S Y K L M F G Q G T S L S V I P	
TRAJ52	P(4)	agtcgttgt	aaaactctccac	tgcaggg	AGTAGCACGGCAGCTCTGAGTTAGAAAAAGAAGGAACTTGACTAACCGTGAAGCCGA	gaagct
					* H G S S E L E K E G T * L T V K P	
TRAJ53	F	ggttcttgt	aaaggcttccat	cgcagtg	TTAATACTGGTGGTGCCTATGGAAAGCTGATGTTTGGACAAGGAACCACTTTGACTGTCCATCCAA	gtaagt
					N T G G A Y G K L M F G Q G T T L T V H P	
TRAJ54	F	cgtttttgt	aaagcctcccct	gactgtg	AGACGAGTAGAGCTAGCAGCTATATACAGACATTTGGAAAAGGCACTCTCTTAATTGTGAATCCAA	gtaagt
					T S R A S S Y I Q T F G K G T L L I V N P	
TRAJ55	F	tttatgtaa	agctctttgctg	gggtgtg	ACTCAGGTGCAGGCCAGGAGCTGGTATTCGGGCGAGGAACCAAGCTGACCATCAACCCCA	gtgagt
					S G A G Q E L V F G R G T K L T I N P	
TRAJ56	F	ggcttttgt	agagcctcgtgt	cattgtg	TAGAACCCCATCCAATAATAAGCTGACATTTGGAAAAGGAACGATCCTGAGTGTTAGACCAG	gtatgt
					R T P S N N K L T F G K G T I L S V R P	
TRAJ57	F	ggtatttgt	aaagcagtctgt	gggggtg	TAACTCAGGACGGATCTGAAAGACTCCTCTTTGGAAAGGGAACGAAGCTGACAGTAAGCCCAC	gtaagt
					T Q D G S E R L L F G K G T K L T V S P	
TRAJ58	F	ggtttttgc	aaagcctcggag	cattgtg	TTCAACAAACCAGTGGCTCTAAGATGATATTTGGGAAAGGGACTCAGCTCACAGTGCAACTTG	gtaagt
					Q Q T S G S K M I F G K G T Q L T V Q L	
TRAJ59	F	agtttatgt	aaaggtgtctgc	tcctgtg	GGAAGCAAGGCAATTTACATTCGGAACCGGGACTCAAGTGAGAGTGAAACTTA	gtaagt
					E A R Q F T F G T G T Q V R V K L	
TRAJ60	F	acttttggt	aaagggcccagg	cactgtg	TAAAGAGCACCGAGAAATTCACCTTTGGGAAAGGGACTCAGTTAATTGTAAGCCTGG	gtgagt
					K S T E K F T F G K G T Q L I V S L	
TRAJ61	P(5)	agtttttgt	aaaggtgttcac	tcctgtg	GGAATGAGGTTAGTGGGAAACTGACATTTTGGAACCAAGACTAGAGGAATCTTGAAACTCA	gtgcgt
					N E V S G K L T F W N Q D * R N L E T	

Notes:

(1)ORF because of LEU instead of J-PHE

(2)ORF because of ALA instead of J-GLY

(3) Pseudogene because of one "N" missing at the end of the J-REGION

(4) Pseudogene because of frameshifts in J-REGION and defective splice site

(5) Pseudogene because of frameshifts in J-REGION

(c)

```
                                                                                    C-DOMAIN
                       A       AB       B          BC           C    CD       D         DE            E      EF   F          FG            G
                    (1-15)          (16-26)     (27-38)    (39-45)       (77-84)                  (85-96)     (97-104)  (105-117)    (118-128)
                ---------------->  ------------>              -------->      -------->              ------------->  --------->  ----------->  ----------->
                1        10  15   16     2326 27        38 3941 45      77 80 84               85  89    96  97    104 105      117 118121
        87654321|........|....|123|......|..| |..........| |.|...|1234567|..|...|12345677654321|...|......|12|......| |...........| |..|.....
TRAC  [EX1] XIKDPNPTVYQLRSPE.......SSNTSVCLFT DFDS.....NQI NLTQIGG.......YEWNMVHKTDSTVLN.MEILGSKSNGIVTWGN......TSDSGC TNTFNE.NIEFVD NF
```

```
                 CONNECTING-REGION          TRANSMEMBRANE-REGION  CYTOPLASMATIC-REGION
                                           |                     |
      [EX2] GIPCDAKLVEKSFET [EX3] DVNLNSQNLSVTVFRILLLKVVGFNLLMTLRLWSS
```
