# Supplementary material for: Sheep (Ovis aries) T cell receptor alpha (TRA) and delta (TRD) genes and genomic organization of the TRA/TRD locus
Source: BMC Genomics. 2015 Sep 18;16:709. doi: 10.1186/s12864-015-1790-z (PMC4574546; doi:10.1186/s12864-015-1790-z)
Supplement: Additional file 7: — Protein display of the TRA cDNA clones. The TRAV and TRAJ genes, named in accordance with the criteria specified in the text, are listed respectively at the left and the right of the figure. The cDNA clones are grouped by TRAV subgroups and those containing the same TRAV gene and allele were boxed and classified with alphabetic letters. Leader region (L-PART1 + L-PART2), CDR-IMGT and FR-IMGT are also indicated, according to the IMGT unique numbering for V-DOMAIN [27]. The five conserved AA of the V-DOMAIN (1st-CYS 23, CONSERVED-TRP 41, hydrophobic AA 89, 2nd-CYS 104 and J-PHE 118) are indicated in bold. The AA changes between genes and allele of the same TRAV subgroup, if any, are shaded. The name of the clones is also reported. (DOC 135 kb) [file 12864_2015_1790_MOESM7_ESM.doc]

TRAV gene	Clone name	L-REGION	A (1-15)	B (16-26)	BC (27-38)	C (39-46)	C' (47-55)	C'C" (56-65)	C" (66-74)	D (75-84)	E (85-96)	F (97-104)	FG (105-117)	G (118-128)	TRAJ gene
			FR1-IMGT (1-26)		CDR1-IMGT (27-38)	FR2-IMGT (39-55)		CDR2-IMGT (56-65)	FR3-IMGT (66-104)				CDR3-IMGT (105-117)	FR4-IMGT (118-128)	
			1 10 15	16 23 26	27 38	3941 46	47 55	56 65	66 74	75 80 84	85 89 96	97 104	105 111 1234321 112 117	118 128	
**TRAV14**															
TRAV14A*01	V14UM2	MLLSSLLRVVVASLCLGSII	AQKVTQDQPQVLVQE	KEAVTLDCKYD	TSDS.....RYS	LLWYKQPS	SGGMILLIR	QDSY..NQQN	ATE.....G	RYSLNFQKAS	KSITLVISAPQL	EDSAVYFC	ALSLSG........NTGRLT	FGQGTVLQVKP	TRAJ37
TRAV14A*01	V14UM5	MLLSSLLRVVVASLCLGSII	AQKVTQDQPQVLVQE	KEAVTLDCKYD	TSDS.....RYS	LLWYKQPS	SGGMILLIR	QDSY..NQQN	ATE.....G	RYSLNFQKAS	KSITLVISAPQL	EDSAVYFC	ALSPGHD.......NSYKLM	FGQGTSLSVIP	TRAJ51
TRAV14A*01	V14UM9	MLLSSLLRVVVASLCLGSII	AQKVTQDQPQVLVQE	KEAVTLDCKYD	TSDS.....RYS	LLWYKQPS	SGGMILLIR	QDSY..NQQN	ATE.....G	RYSLNFQKAS	KSITLVISAPQL	EDSAVYFC	ALSLFQ.........ASQLN	FGTGTRLTITA	TRAJ49
TRAV14A*01	V14UM8	MLLSSLLRVVVASLCLGSII	AQKVTQDQPQVLVQE	KEAVTLDCKYD	TSDS.....RYS	LLWYKQPS	SGGMILLIR	QDSY..NQQN	ATE.....G	RYSLNFQKAS	KSITLVISAPQL	EDSAVYFC	AFLELN........AGYVLH	FGQGTSVLVTP	TRAJ41
TRAV14A*01	V14UM1	MLLSSLLRVVVASLCLGSII	AQKVTQDQPQVLVQE	KEAVTLDCKYD	TSDS.....RYS	LLWYKQPS	SGGMILLIR	QDSY..NQQN	ATE.....G	RYSLNFQKAS	KSITLVISAPQL	EDSAVYFC	ALRPYS........GNYKYV	FGAGTKLQVLT	TRAJ40
TRAV14A*01	V14UT3	MLLSSLLRVVVASLCLGSII	AQKVTQDQPQVLVQE	KEAVTLDCKYD	TSDS.....RYS	LLWYKQPS	SGGMILLIR	QDSY..NQQN	ATE.....G	RYSLNFQKAS	KSITLVISAPQL	EDSAVYFC	ALSSLWG......GGSERLL	FGKGTKLTVSP	TRAJ57
TRAV14A*01	V14UT12	MLLSSLLRVVVASLCLGSII	AQKVTQDQPQVLVQE	KEAVTLDCKYD	TSDS.....RYS	LLWYKQPS	SGGMILLIR	QDSY..NQQN	ATE.....G	RYSLNFQKAS	KSITLVISAPQL	EDSAVYFC	ASTQGG.........SERLL	FGKGTKLTVSP	TRAJ57
TRAV14A*01	V14UT10	MLLSSLLRVVVASLCLGSII	AQKVTQDQPQVLVQE	KEAVTLDCKYD	TSDS.....RYS	LLWYKQPS	SGGMILLIR	QDSY..NQQN	ATE.....G	RYSLNFQKAS	KSITLVISAPQL	EDSAVYFC	ALSEPIR......GAYGKLM	FGQGTTLTVHP	TRAJ53
TRAV14A*01	V14UT7	MLLSSLLRVVVASLCLGSII	AQKVTQDQPQVLVQE	KEAVTLDCKYD	TSDS.....RYS	LLWYKQPS	SGGMILLIR	QDSY..NQQN	ATE.....G	RYSLNFQKAS	KSITLVISAPQL	EDSAVYFC	ALSEPVA.......SSYIQT	FGKGTLLIVNP	TRAJ54
TRAV14A*02	V14UT8	MLLSSLLRVVVASLCLGSII	AQKVTQDQPQVLGQE	KEAVTLDCKYD	TSDS.....RYS	LLWYKQPS	SGGMILLIR	QDSY..NQQN	ATE.....G	RYSLNFQKAS	KSITLVISAPQL	EDSAVYFC	ALSEPGS.......GYNTLT	FGKGTVLLVFP	TRAJ12
TRAV14A*02	V14UT2	MLLSSLLRVVVASLCLGSII	AQKVTQDQPQVLGQE	KEAVTLDCKYD	TSDS.....RYS	LLWYKQPS	SGGMILLIR	QDSY..NQQN	ATE.....G	RYSLNFQKAS	KSITLVISAPQL	EDSAVYFC	ALSAHNT......GSGGKVI	FGTGTRLQVTL	TRAJ44
TRAV14B*01	V14UM11	MLLSSLLRVVVASLCLGSII	AQKVTQDQPQVLVQE	KEAVTLDCKYD	TSES.....RYS	LLWFKQPS	SGGMIFLIR	QDSY..NQQN	ATE.....G	RYSLNFQKAS	KSITLDISASQL	EDSAVYFC	ALPGKQ........TTGKIV	FGRGTQLHVLP	TRAJ30
TRAV14B*01	V14UT4	MLLSSLLRVVVASLCLGSII	AQKVTQDQPQVLVQE	KEAVTLDCKYD	TSES.....RYS	LLWFKQPS	SGGMIFLIR	QDSY..NQQN	ATE.....G	RYSLNFQKAS	KSITLDISASQL	EDSAVYFC	ALSECQP.......GTGKLT	FGDGTALTVKP	TRAJ27
TRAV14C*01	V14UM6	MLLSSLLRVVVASLCLGSSI	AQKVTQDQPPMSVQE	KETVTLDCTYD	TSIT.....TYS	LFWYKQPS	SGVMTFLIR	QDSS..NKPN	ATE.....G	RYSLNFQKAS	KFITLTISASQL	EDSAVYFC	ALEGRG.........NYKYV	FGAGTKLQVLT	TRAJ40
TRAV14C*01	V14UT5	MLLSSLLRVVVASLCLGSSI	AQKVTQDQPPMSVQE	KETVTLDCTYD	TSIT.....TYS	LFWYKQPS	SGVMTFLIR	QDSS..NKPN	ATE.....G	RYSLNFQKAS	KFITLTISASQL	EDSAVYFC	ALSDPRSF....LNAGYVLH	FGQGTSVLVTP	TRAJ41
TRAV14C*01	V14UT6	MLLSSLLRVVVASLCLGSSI	AQKVTQDQPPMSVQE	KETVTLDCTYD	TSIT.....TYS	LFWYKQPS	SGVMTFLIR	QDSS..NKPN	ATE.....G	RYSLNFQKAS	KFITLTISASQL	EDSAVYFC	ALSEPNVG.....SSQGKLI	FGKGTMVSVKP	TRAJ42
**TRAV21**															
TRAV21A*01	V21UT14	MDTSLSLLILWLQLDWSSS	KQDVSQSPEALSVRE	GDSLVLNCSYT	DSA......LYF	LQWFRQDP	GKGLISLLS	IQAN...QKE	QTS.....G	RITVSLDKSS	RHSALYIATSQH	SDSTTYLC	AVSLLNS.......GYNTLT	FGKGTVLLVFP	TRAJ12
TRAV21A*02	V21UM1/T3	MDTSLSLLILWLQLDWASS	KQDVSQSPEALSVRE	GDSLVLNCSYT	DSA......LYF	LQWFRQDP	GKGLISLLS	IQAN...QKE	QTS.....G	RITVSLDKSS	RHSALYIATSQR	SDSTTYLC	AVRRGG.........NYKYV	FGAGTKLQVLT	TRAJ40
TRAV21A*02	V21UM13	MDTSLSLLILWLQLDWASS	KQDVSQSPEALSVRE	GDSLVLNCSYT	DSA......LYF	LQWFRQDP	GKGLISLLS	IQAN...QKE	QTS.....G	RITVSLDKSS	RHSALYIATSQR	SDSTTYLC	AVEEGT........GGFKVV	FGTGTKLFVET	TRAJ9
TRAV21A*02	V21UT13	MDTSLSLLILWLQLDWASS	KQDVSQSPEALSVRE	GDSLVLNCSYT	DSA......LYF	LQWFRQDP	GKGLISLLS	IQAN...QKE	QTS.....G	RITVSLDKSS	RHSALYIATSQR	SDSTTYLC	AAGTGG........VMSKLT	FGKGTQVSIIS	TRAJ2
TRAV21A*02	V21UM2	MDTSLSLLILWLQLDWASS	KQDVSQSPEALSVRE	GDSLVLNCSYT	DSA......LYF	LQWFRQDP	GKGLISLLS	IQAN...QKE	QTS.....G	RITVSLDKSS	RHSALYIATSQR	SDSTTYLC	AESGS..........GDRLT	FGTGTRLAVRP	TRAJ47
TRAV21A*02	V21UM9	MDTSLSLLILWLQLDWASS	KQDVSQSPEALSVRE	GDSLVLNCSYT	DSA......LYF	LQWFRQDP	GKGLISLLS	IQAN...QKE	QTS.....G	RITVSLDKSS	RHSALYIATSQR	SDSTTYLC	AVGRA..........YGKLM	FGQGTTLTVHP	TRAJ53
TRAV21A*02	V21UM5	MDTSLSLLILWLQLDWASS	KQDVSQSPEALSVRE	GDSLVLNCSYT	DSA......LYF	LQWFRQDP	GKGLISLLS	IQAN...QKE	QTS.....G	RITVSLDKSS	RHSALYIATSQR	SDSTTYLC	AVYQGG.........SERLL	FGKGTKLTVSP	TRAJ57
TRAV21A*02	V21UM6	MDTSLSLLILWLQLDWASS	KQDVSQSPEALSVRE	GDSLVLNCSYT	DSA......LYF	LQWFRQDP	GKGLISLLS	IQAN...QKE	QTS.....G	RITVSLDKSS	RHSALYIATSQR	SDSTTYLC	AVRPSRT.......SSYIQT	FGKGTLLIVNP	TRAJ54
TRAV21B*01	V21UT10	MDTSLSLLILWLQLDWASS	KQDASQSPEALRARE	GEGLVLNCSHT	DRA......LYF	LQWFRQDP	GKGPASLLS	IQAN...QKE	QAR.....G	RITVSLDKSS	RHSALYIATSQH	SDSTTYLC	ARNT............NRFY	FGSGTKLSVKP	TRAJ21
TRAV21B*01	V21UT12	MDTSLSLLILWLQLDWASS	KQDASQSPEALRARE	GEGLVLNCSHT	DRA......LYF	LQWFRQDP	GKGPASLLS	IQAN...QKE	QAR.....G	RITVSLDKSS	RHSALYIATSQH	SDSTTYLC	AVKGG..........GYTWT	FGSGTRLLVRP	TRAJ13
TRAV21C*01	V21UM10	MDTSLSLLILWLQLDWVSS	KQDVSQSPEALSVRE	GDSLVLNCSYT	DSA......LYF	LQWFRQDP	AKGPASLLS	IQAN...QKE	QAR.....G	RITVSLDKSS	RHSALYIATSQH	SDSTTYLC	AVRFNS.........YGQNY	FGRGTSLTVIP	TRAJ50
TRAV21C*01	V21UT11	MDTSLSLLILWLQLDWVSS	KQDVSQSPEALSVRE	GDSLVLNCSYT	DSA......LYF	LQWFRQDP	AKGPASLLS	IQAN...QKE	QAR.....G	RITVSLDKSS	RHSALYIATSQH	SDSTTYLC	AVRGQT.........TGKIV	FGRGTQLHVLP	TRAJ3
**TRAV25**															
TRAV25A*01	V25UM4	MLLLAPVLILWIQISEMN	GQQIKHFPEFLLLQE	GENFTTYCNSS	STF.......YN	LQWYKQRP	GGSPVFLMI	LTKP...GEA	KTE.....Q	RLTGWLGESR	QHSSLHLAAAQL	SDAGTYFC	AERDA..........GNVFT	FGGGTRLMVKP	TRAJ39
TRAV25A*01	V25UM15	MLLLAPVLILWIQISEMN	GQQIKHFPEFLLLQE	GENFTTYCNSS	STF.......YN	LQWYKQRP	GGSPVFLMI	LTKP...GEA	KTE.....Q	RLTGWLGESR	QHSSLHLAAAQL	SDAGTYFC	AYWN.............TLI	FGSGTTLTVKP	TRAJ4
TRAV25A*02	V25UM5	MLLLAPVLILWIQISEMN	GQQIKHFSEFLLLQE	GENFTTYCNSS	STF.......YN	LQWYKQRP	GGSPVFLMI	LTKP...GEA	KTE.....Q	RLTGQLGESR	QHSSLHLAAAQL	SDAGSYFC	ARLDY..........NNDLR	FGAGTRLMVKP	TRAJ43
TRAV25A*02	V12UT13	MLLLAPVLILWIQISEMN	GQQIKHFSEFLLLQE	GENFTTYCNSS	STF.......YN	LQWYKQRP	GGSPVFLMI	LTKP...GEA	KTE.....Q	RLTGQLGESR	QHSSLHLAAAQL	SDAGSYFC	ARVGGA.........GNALV	FGKGTTVSVHP	TRAJ16
TRAV25B*01	V25UM2	MLLLAPVLILWIQISEMN	GKQIKHFPEFLLLQE	GENFTTYCNSS	STF.......YS	LQWYKQRP	GGSPVFLMI	LTKP...GEA	KTE.....Q	RLTGRFGESR	QHSSLHLAAAQL	SDAGTYFC	AGQNTG........GGNRLI	FGKGTQLIIQP	TRAJ46
TRAV25B*01	V25UM3	MLLLAPVLILWIQISEMN	GKQIKHFPEFLLLQE	GENFTTYCNSS	STF.......YS	LQWYKQRP	GGSPVFLMI	LTKP...GEA	KTE.....Q	RLTGRFGESR	QHSSLHLAAAQL	SDAGTYFC	AGNS............NKLI	FGAGTRLQVFP	TRAJ34
TRAV25C*01	V25UM6	MLLLAPVLILWIQISEMN	GQQIKHFPEFLLLQE	GENFTTYCNSS	STF.......LH	LQWYKQSP	GGSPVLLMI	LAKA...GEV	KTE.....Q	RLTGRLGETR	QHSSLHLAAAQL	SDAGTYFC	AGNN............YKFT	FGSGTTVTVRA	TRAJ20
TRAV25C*01	V25UT15	MLLLAPVLILWIQISEMN	GQQIKHFPEFLLLQE	GENFTTYCNSS	STF.......LH	LQWYKQSP	GGSPVLLMI	LAKA...GEV	KTE.....Q	RLTGRLGETR	QHSSLHLAAAQL	SDAGTYFC	AEKST...........EKFT	FGKGTQLIVSL	TRAJ60
TRAV25C*01	V25UT14	MLLLAPVLILWIQISEMN	GQQIKHFPEFLLLQE	GENFTTYCNSS	STF.......LH	LQWYKQSP	GGSPVLLMI	LAKA...GEV	KTE.....Q	RLTGRLGETR	QHSSLHLAAAQL	SDAGTYFC	ATQGG..........SERLL	FGKGTKLTVSP	TRAJ57
TRAV25D*01	V25UT7	MLLIAPVLILWMQIPQMN	GQQIKHFPEFLLLQE	GENFTTYCNFS	STF.......YN	LQWYKQRP	GGSPVLLMI	LAKG...GEV	KTQ.....Q	RLTVRFGESR	QHSSLHLTATQP	SDVGTYFC	AFSAG...........YVLH	FGQGTSVLVTP	TRAJ41
TRAV25E*01	V25UM8	MLLIAPVLILWMQIPQVN	AQQISQIPQFLLLQE	GENFTTYCNSS	STF.......YS	LQWYKQSP	GGSPVLLMI	LAKG...GEV	KTE.....Q	RLTGRLGETR	KHGSLHLAAAQL	SDAGTYFC	AGWTG..........GFKVV	FGTGTKLFVET	TRAJ9
TRAV25E*01	V25UM7	MLLIAPVLILWMQIPQVN	AQQISQIPQFLLLQE	GENFTTYCNSS	STF.......YS	LQWYKQSP	GGSPVLLMI	LAKG...GEV	KTE.....Q	RLTGRLGETR	KHGSLHLAAAQL	SDAGTYFC	AQTSG...........SKMI	FGKGTQLTVQL	TRAJ58
TRAV25F*01	V25UT12	MLLIAPVLILWMQIPQVN	AQQISQIPQFLFLQE	GENFTTYCNSS	STL.......TN	LQWYKQRP	GESPVRLMI	LAKA...GEV	KTE.....Q	RLTGRLGDTR	KHGSLHLAAAQL	SDAGTYFC	AGKPS..........NNKLT	FGKGTILSVRP	TRAJ56
TRAV25F*01	V25UM9	MLLIAPVLILWMQIPQVN	AQQISQIPQFLFLQE	GENFTTYCNSS	STL.......TN	LQWYKQRP	GESPVRLMI	LAKA...GEV	KTE.....Q	RLTGRLGDTR	KHGSLHLAAAQL	SDAGTYFC	AGGGS..........GDRLT	FGTGTRLAVRP	TRAJ47
TRAV25F*01	V25UT8	MLLIAPVLILWMQIPQVN	AQQISQIPQFLFLQE	GENFTTYCNSS	STL.......TN	LQWYKQRP	GESPVRLMI	LAKA...GEV	KTE.....Q	RLTGRLGDTR	KHGSLHLAAAQL	SDAGTYFC	AGGGCG........SGDRLT	FGTGTRLAVRP	TRAJ47
TRAV25G*01	V25UM1	MLLIARV*LH*LILWMQIPQVN	AQQISQIPQFLLQQE	GKNFTTYCNSS	STF.......YS	LQWYKQSP	GGSPVLLMI	LAKA...GEV	KTE.....Q	RLTGRLGETM	KHGSLHLAAAQL	SDAGTYFC	TRLNY..........NNDLR	FGAGTRLMVKP	TRAJ43
TRAV25G*01	V25UM10	MLLIARV*LH*LILWMQIPQVN	AQQISQIPQFLLQQE	GKNFTTYCNSS	STF.......YS	LQWYKQSP	GGSPVLLMI	LAKA...GEV	KTE.....Q	RLTGRLGETM	KHGSLHLAAAQL	SDAGTYFC	TGRTF..........GNVLH	WGSGTQVIVIP	TRAJ35
TRAV25G*01	V25UM11	MLLIARV*LH*LILWMQIPQVN	AQQISQIPQFLLQQE	GKNFTTYCNSS	STF.......YS	LQWYKQSP	GGSPVLLMI	LAKA...GEV	KTE.....Q	RLTGRLGETM	KHGSLHLAAAQL	SDAGTYFC	TLNSY...........GQNY	FGRGTSLTVIP	TRAJ50
TRAV25G*01	V25UT6	MLLIARV*LH*LILWMQIPQVN	AQQISQIPQFLLQQE	GKNFTTYCNSS	STF.......YS	LQWYKQSP	GGSPVLLMI	LAKA...GEV	KTE.....Q	RLTGRLGETM	KHGSLHLAAAQL	SDAGTYFC	TDSSY...........GQNY	FGRGTSLTVIP	TRAJ50
