# Supplementary material for: Sheep (Ovis aries) T cell receptor alpha (TRA) and delta (TRD) genes and genomic organization of the TRA/TRD locus
Source: BMC Genomics. 2015 Sep 18;16:709. doi: 10.1186/s12864-015-1790-z (PMC4574546; doi:10.1186/s12864-015-1790-z)
Supplement: Additional file 8: — Corrispondence between ovine and bovine germline TRAV repertoires. The bovine TRAV gene sequences were retrieved by Connelley et al. [16]. Correspondence was established as reported in Methods. (DOC 59 kb) [file 12864_2015_1790_MOESM8_ESM.doc]

**Additional file 8.** Corrispondence between ovine and bovine germline TRAV repertoires

| **Sheep** | **Cow** |
| --- | --- |
| TRAV1 | TRAV1 |
| TRAV2 | TRAV2 |
| TRAV3 | TRAV3 |
| TRAV4 | TRAV4 |
| TRAV5 | TRAV5 |
| TRAV6 | TRAV6 |
| TRAV8 | TRAV8i |
| TRAV9 | TRAV9 |
| TRAV10 | TRAV10 |
| - | TRAV11 |
| TRAV12 | TRAV12 |
| TRAV13 | TRAV13 |
| TRAV14 | TRAV14 |
| TRAV16 | TRAV16 |
| TRAV17 | TRAV17 |
| TRAV18 | TRAV18 |
| - | TRAV19 |
| - | TRAV20 |
| TRAV21 | TRAV21 |
| TRAV22 | TRAV22 |
| TRAV23 | TRAV23 |
| - | TRAV24 |
| TRAV25 | TRAV25 |
| TRAV26 | - |
| TRAV27 | TRAV27 |
| TRAV28 | TRAV28 |
| TRAV29 | TRAV29 |
| - | TRAV33 |
| TRAV34 | TRAV34 |
| TRAV35 | TRAV35 |
| TRAV36 | TRAV36 |
| TRAV37 | TRAV37 |
| TRAV38 | TRAV38 |
| TRAV39 | TRAV39 |
| TRAV41 | TRAV41 |
| TRAV42 | TRAV8f-g-j-l-n |
| TRAV43 | TRAVY |
| TRAV44 | TRAV26 |
| TRAV45 | TRAVX |

The bovine TRAV gene sequences were retrieved by Connelley et al. [16]. Correspondence was established as reported in Methods.
